# Ring Strain Energies in Four-Membered Heterocycles Containing an Element of Groups 13–16

**DOI:** 10.1021/acs.inorgchem.5c04569

**Published:** 2025-12-09

**Authors:** Alicia Rey Planells, Antonio García Alcaraz, Arturo Espinosa Ferao

**Affiliations:** † Departamento de Química Orgánica, Facultad de Química, 16751 Universidad de Murcia, Campus de Espinardo, 30071 Murcia, Spain; ‡ Faculty of Pharmacy, University of Castilla-La Mancha, Calle Almansa 14−Edif. Bioincubadora, 02008 Albacete, Spain

## Abstract

The ring strain energy (RSE) of four-membered rings (4MRs) incorporating only one element from groups 13–16, exhibit moderate values of approximately 25 kcal·mol^–1^ for compounds containing second-row elements, and decreasing to 14–19 kcal·mol^–1^ as one progresses down the periodic table. 4MRs containing Bi, S, Se, Te and Po, exhibited higher RSEs than their three-membered analogues, which seems to be related to enhanced σ-antiaromaticity. The relationship between RSEs and some structural and electronic parameters was investigated. The strain-relieving effect observed with an increase in heteroatom size in all groups, may also coexist with that provided by the increase in the p-character of the AO used by the heteroatom in groups 15 and 16 in the endocyclic bonds. Furthermore, the RSE is found to correlate linearly with the C–Z–C bond angles and Z–C bond distances, as well as with the corresponding relaxed force constants. There is also a strong correlation between the RSEs of 4MRs with elements of groups 15 and 16 and the HOMO–LUMO gaps, as well as with the electron density and its Laplacian, in the ring critical points. In addition, an effective additive method for rapid estimation of RSEs based on ring atoms or endocyclic bonds contributions is disclosed.

## Graphical abstract

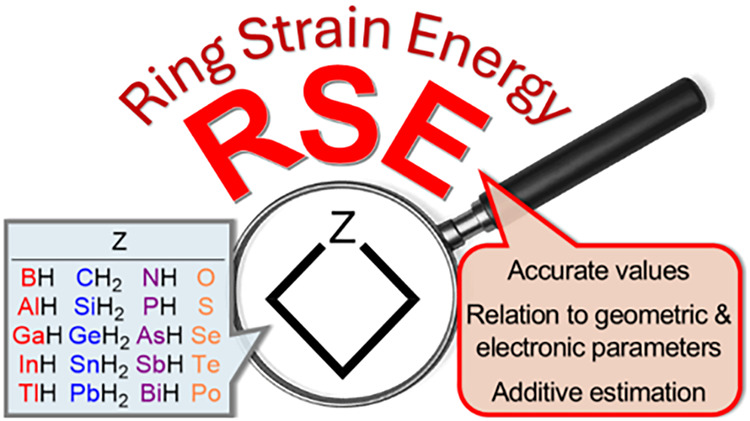

## Introduction

Ring strain energy (RSE) is a defining characteristic of small cyclic systems,^1^ where the internal bond angles are compressed, and the magnitudes deviate significantly from the ideal “unstrained” values observed in acyclic analogue systems.^2^ This is the result of a combination of different strains, namely angular (also known as Baeyer strain),^3^ torsional (also known as eclipsing or Pitzer strain) and transannular (known as van der Waals strain).^4^ The consequence of this surplus internal energy is the release of energy in combustion reactions.^5^ Collectively, these characteristics can be utilized as a driving force in ring-opening reactions (ROR) and ring-opening polymerizations (ROP). Consequently, the calculation of RSEs has become a significant area of focus,^6^ with considerable computational resources dedicated to estimating RSEs of both organic^7^ and inorganic^8,9^ rings.

In order to calculate RSEs using a computational method, it is necessary to ensure that the chemical reactions are properly balanced. In this approach, one of the reactants is the strained cyclic molecule, while the products are unstrained acyclic species. This ensures that all energetic effects are compensated on both sides of the equation, with the exception of the RSE itself. By employing hybridization, electron pair counting and bonding criteria,^10^ the equations can be categorized into various groups, including isodesmic,^11,12^ homodesmotic^13,14^ and hyperhomodesmotic.^15,16^

The smallest-sized cyclic molecules are three-membered rings (3MRs), which should have the highest ring strain energy. The structure and high reactivity of these compounds have long been a source of fascination for the chemical community, from both a theoretical and experimental standpoint..^11,12,17–19^ A series of comprehensive theoretical studies on the RSE of model saturated 3MRs containing elements from groups 13, 14, 15, and 16 have been recently published.^20^ This has been complemented by a similar study on the simplest unsaturated rings containing only one heteroatom.^21^ These studies compare different methods to determine the most effective RSE values. Trends in these values across groups and periods are also analyzed, as well as the relationship between RSEs and various electronic and geometrical parameters. Additionally, the RSEs of other 3MRs were examined.^22–24^

The next in size to three-membered rings are four-membered rings (4MRs), also having great importance in a number of areas of chemistry. Despite their larger size, four-membered rings also exhibit high RSEs, making them an intriguing synthetic target. However, there is currently a gap in the literature regarding the RSE of four-membered heterocycles containing elements from groups 13, 14, 15, and 16. A particularly relevant class of four-membered rings is that of β-lactams, due to their pivotal role in medicinal chemistry and the ongoing challenge of antibiotic resistance.^25–27^ Their pronounced reactivity, stemming from significant ring strain, is central to their biological function and synthetic utility, thus remaining a key structural motif in the study of strained small-ring systems and their associated reactivity. Also, derivatives of the related cyclobutanone ring have paramount importance not only in synthesis, but also due to biomedical^28–33^ and material science^34–36^ applications.

The objective of this study is to conduct an in-depth analysis of the ring strain energies for the heterocyclic 4MRs model **1** ^**El**^ (CH_2_)_3_Z. This model features an element (El) from groups 13–16 with its characteristic valences (3, 4, 3, and 2 for groups 13, 14, 15 and 16, respectively), along with hydrogen atom bonds (Figure 1). Furthermore, the research will examine and evaluate the potential correlation between various electronic and geometrical parameters and the calculated RSEs. An additive method for the fast estimation of RSE is also proposed, including as well the cyclobutanone, β-lactam and 1,2-oxa- and -thiaphosphetane ring systems.

**Figure 1:**
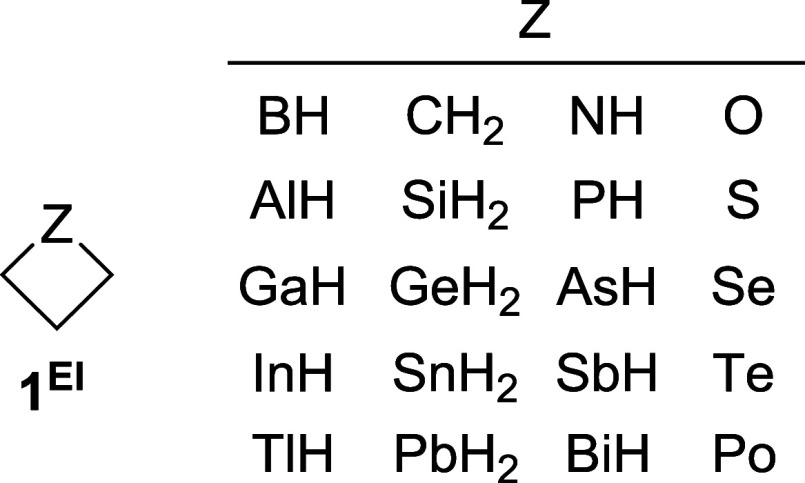
Four-membered heterocycles **1** ^**El**^ studied in this work.

## Results and Discussion

### I Ring Strain Energy Values

To ensure the accuracy of the RSE values, the most appropriate homodesmotic reactions were selected (Figure 2). Homodesmotic reactions are considered the second most accurate in a broad hierarchy of reactions used for thermochemical evaluations, due to the conservation of large fragments.^10^ The study performed on 3MRs demonstrated that the RSE values obtained through homodesmotic reactions are practically identical to those obtained with hyperhomodesmotic reactions, the first ranked type in quality and accuracy. Therefore, it is not cost-effective to use the latter due to their higher computational cost.^20^ Furthermore, hyperhomodesmotic ring-opening reactions result in the formation of longer-chain acyclic products, where interactions frequently occur between groups that are far apart in the molecular connectivity but spatially close within the chain. This can lead to the introduction of undesired uncompensated energy terms, which may introduce some degree of error.

**Figure 2:**
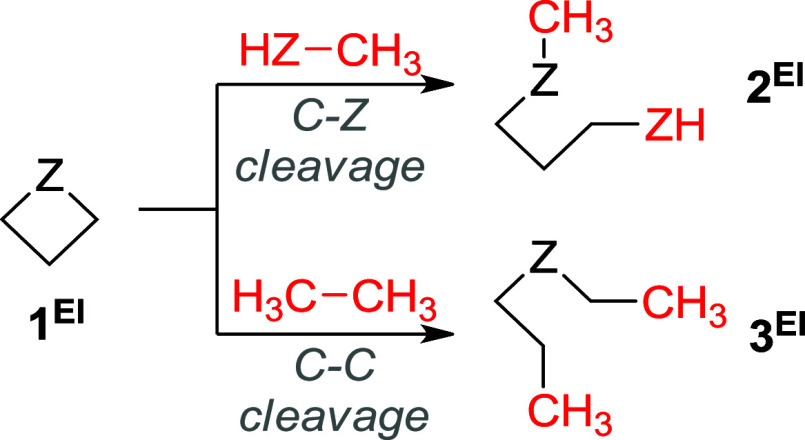
Homodesmotic reactions used for the estimation of the RSEs of **1** ^**El**^ derivatives.

The results (Table 1) demonstrate that the heterocycles of groups 13 and 14 exhibit higher RSEs than those of groups 15 and 16, a phenomenon that is also observed in the 3MRs.^20^ It is crucial to highlight that, across all groups 13–16, the heterocycles exhibiting the greatest strain are those containing the smallest sized second-row heteroelements and the RSE generally decreases as one moves down through the groups. However, the observed trend in groups 13 and 14, whereby RSE decreases on moving down through the group, is opposite to that seen in 3MRs and presents an anomaly in the third period. This is to some extent attributable to the electronegativities of the second-period elements. Therefore, both aluminum (χ^Pauling^ = 1.61) and silicon (χ^Pauling^ = 1.90) are more electropositive than the third period elements, namely gallium (χ^Pauling^ = 1.81) and germanium (χ^Pauling^ = 2.01), due to the contraction of the d-block in the latter two elements. This results in a less acidic character and more covalent bonds in Ga and Ge atoms compared to Al and Si. Therefore, both aluminum and silicon are exceptions to the general trend.^22–24^

**Table 1:** Ring Strain Energies (kcal·mol^–1^) Computed [DLPNO/CCSD­(T)/def2-QZVPP­(ecp)] for Compounds **1** ^**El**^ ^b^ ^20^

group 13	group 14	group 15	group 16
B 26.49 (38.66)	C 26.43 (27.86)	N 25.84 (27.37)	O 25.00 (26.55)
Al 21.36 (44.42)	Si 23.16 (36.63)	P 19.38 (20.38)	S 19.42 (17.87)
Ga 23.52 (43.84)	Ge 23.24 (36.40)	As 17.91 (18.23)	Se 17.99 (16.13)
In 20.65 (^a^)	Sn 21.29 (35.79)	Sb 16.19 (16.23)	Te 16.42 (13.76)
Tl 17.58 (^a^)	Pb 19.15 (^a^)	Bi 14.48 (12.55)	Po 15.19 (11.45)

a These elements were reported not to form proper 3MRs.^20^

b In parentheses the values reported for the 3MR analogues at the same level of theory.^20^

It is also noteworthy that some 4MRs, specifically those of Bi, S, Se, Te and Po, exhibit a higher RSE than their respective homologous 3MRs (Table 1). This finding is somewhat counterintuitive, as one would expect that the smaller a heterocycle is, the higher its RSE due to higher due to higher bond angle strain. The rationale for this phenomenon can be traced back to the stabilization of 3MRs, which is attributed to their σ-aromaticity, and the concurrent destabilization of 4MRs, which can be ascribed to σ-antiaromaticity.^37,38^ The aforementioned phenomena can be ascribed to the simple electron count of six or eight electrons in a set of three or four skeletal σ-type molecular orbitals in 3MRs or 4MRs, respectively. The observed inversion in their RSE can be tentatively explained by an unequal extent of stabilizing σ-aromaticity^8^ and destabilizing σ-antiaromaticity along the p-block, especially for this ’right-bottom corner’, consistent with the characteristic σ-ring current behavior reported for small saturated rings (see the SI. for a qualitative MO analysis and effect of heaviest lone pair-containing heteroelements). Despite the little σ-aromatic stabilization reported for cyclopropane,^39^ some magnetic criteria, such as the axial component of the magnetic shielding tensor (σ_ZZ_), provide a complementary perspective, probing the response of the σ-electron system to an external field largely independent of ring strain. Moreover, by evaluating σ_ZZ_(1), 1 Å away from the ring centroid (averaging above and below the plane), the criticized contamination by local in-plane effects is minimized. Indeed, a slight increase in diatropicity for 3MRs, together with a drastic increase in paratropicity for 4MRs, is observed for the heavier pnictogens and chalcogens when σ_ZZ_(1) is compared (Figure 3). As a result, very large σ_ZZ_(1) variation is observed for those ring couples (3MRs/4MRs) having negative variation of RSE (Figure S1).

**Figure 3:**
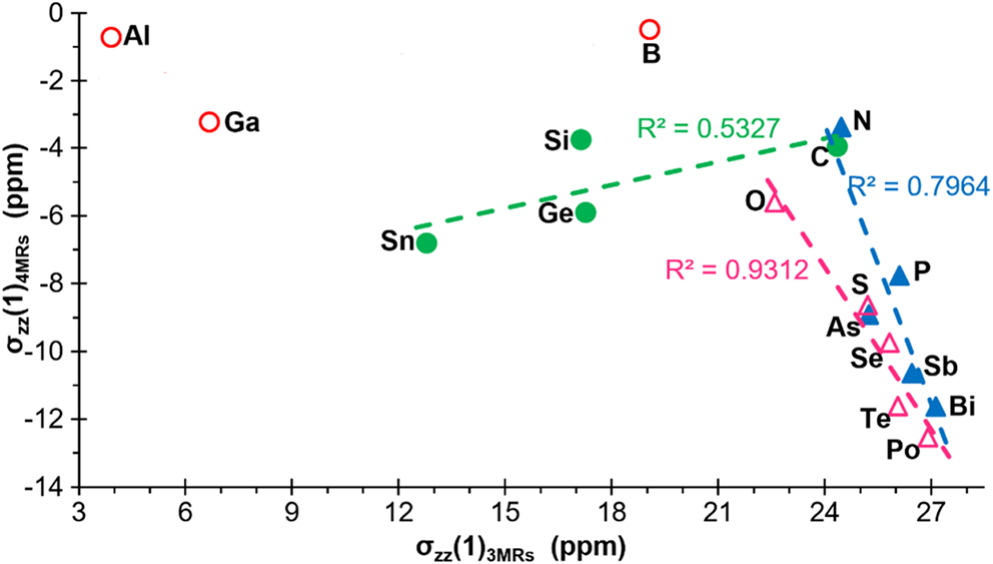
σ_ZZ_(1) of compounds **1** ^**El**^, compared to the corresponding 3MRs, for ‘El’ elements belonging to groups 13 (empty red circle), 14 (filled green circle), 15 (filled blue triangle) and 16 (empty fuchsia triangle).

A comparison is impossible for the most metallic elements as three-membered analogues of **1** ^**In**^, **1** ^**Tl**^ and **1** ^**Pb**^ were reported not to form proper rings, but Dewar-Chatt-Duncanson complexes instead.^20^

### II Relationship of RSEs to Geometric Parameters

In order to elucidate the genesis of the diverse observed RSE trends, a series of factors that may influence RSE were subjected to analysis. Initially, the relaxed bond strength constants and bond angles of both endo- and exocyclic bonds were evaluated.

#### II.1 Endocyclic Bond Angles

One of the most directly relevant properties to the RSE is the force constant associated with the endocyclic bond angles, specifically the C–Z–C, C–C-Z, and C–C–C bond angles for the family of 4MRs under consideration. This can be explained by the fact that, in acyclic species, the greater the stiffness of an endocyclic bond angle, the higher the energy required to compress it to the angle value of one of the corresponding 4MR species studied here, and therefore to a higher ring strain. In C–Z–C bond angles, however, this need not necessarily be the case, since in acyclic species they are already very close to the typical 90–100° angles of the four-membered cycle, especially in heavy atoms. In other words, the concept of ring strain can be understood as the destabilization of endocyclic bond angles in comparison to the reference values observed in analogous acyclic species. To illustrate, the C–C–C angle in propane (calculated at 109.5°) is destabilized by 15.04 kcal·mol^–1^ when compressed to 90°.^20^ Similarly, dimethyl ether is destabilized by 15.69 kcal·mol^–1^ when the equilibrium geometry of the C–O–C angle (111.6°) is compressed to 90°. Therefore, based on the aforementioned destabilization of 0.7713 and 0.7264 kcal mol^–1^ per degree for the C–C–C and C–O–C angles, respectively, it can be extrapolated that the expected ring strain in cyclopropane and oxirane due to compression of the bond angle to approximately 60° should be approximately 38.2 and 37.5 kcal·mol^–1^, respectively. This is considerably higher than the calculated values, mostly due to the above-mentioned σ-aromaticity stabilization in 3MRs.^8^ Nevertheless, the equilibrium bond angles in acyclic species are typically markedly disparate from those observed in small heterocycles, leading to notable variations in the endocyclic angles. This is attributed to the participation of atomic orbitals with distinct hybridization in the aforementioned cyclic and acyclic species. It is therefore of interest to examine the force constants associated with the bond angles in the heterocycles under consideration. In order to prevent contamination of the results by the vibrational modes of other bonds or bond angles within the same molecule, instead of collecting the force constants directly extracted from the frequency calculations, the so-called ‘relaxed force constants’ *k* ^0^ were employed, as they are typically stable and transferable parameters.^40–42^ The mathematical transformation of the Hessian matrix obtained in nonredundant internal coordinates into its inverse (or Moore-Penrose pseudoinverse) gives rise to the matrix *C_ij_*. The inverse values of the diagonal elements yield the relaxed force constants, *k*_ii_^0^ = 1/*C*_ii_.

Initially, the RSEs of the various groups (13, 14, 15 and 16) were plotted against the relaxed force constants of the Z–C–C bond angles (Figure 4). The removal of the lightest element (boron) from the representation for group 13 yielded an acceptable correlation with a positive slope (*R* ^2^ = 0.9275). In contrast, the removal of the lightest element from group 14 (carbon) did not result in an enhanced correlation, which still exhibits a markedly positive slope (*R* ^2^ = 0.4329). Moreover, the relaxed force constant for the heavy tetrel elements exhibits fundamental invariance (approximately 2.4 mdyn·Å). Once more, the exclusion of the lightest elements from the representation of groups 15 and 16 yielded highly robust correlations with positive slopes (*R* ^2^ = 0.9952 and 0.9959, respectively). This proportionality is logical, given that an increase in the value of *k* ^0^ corresponds to a greater difficulty in compressing the bond angle, which in turn results in a higher RSE, as previously discussed. A similar outcome is observed when plotting the RSEs against the relaxed force constants of the C–C–C bond angles (Figure 4b). In this instance, all the lightest elements deviate slightly, and thus have been excluded from the correlations, which yielded excellent results (R^2^ = 0.8839, 0.8923, 0.9824, and 0.9554 for groups 13, 14, 15 and 16, respectively), with a positive slope. In contrast, the relaxed force constants of the C-Z-C bond angles exhibited an inverse trend, as evidenced by a negative slope (Figure 4c). This finding aligns with the observations made in the related study on 3MRs.^20^ For group 13, the elimination of the lightest element does not yield a satisfactory correlation (*R* ^2^ = 0.5261), while for group 14, the correlation improves only marginally (*R* ^2^ = 0.8646). Conversely, the removal of the lightest elements from groups 15 and 16 resulted in the generation of somewhat superior correlations (*R* ^2^ = 0.9925 and 0.9998, respectively).

**Figure 4:**
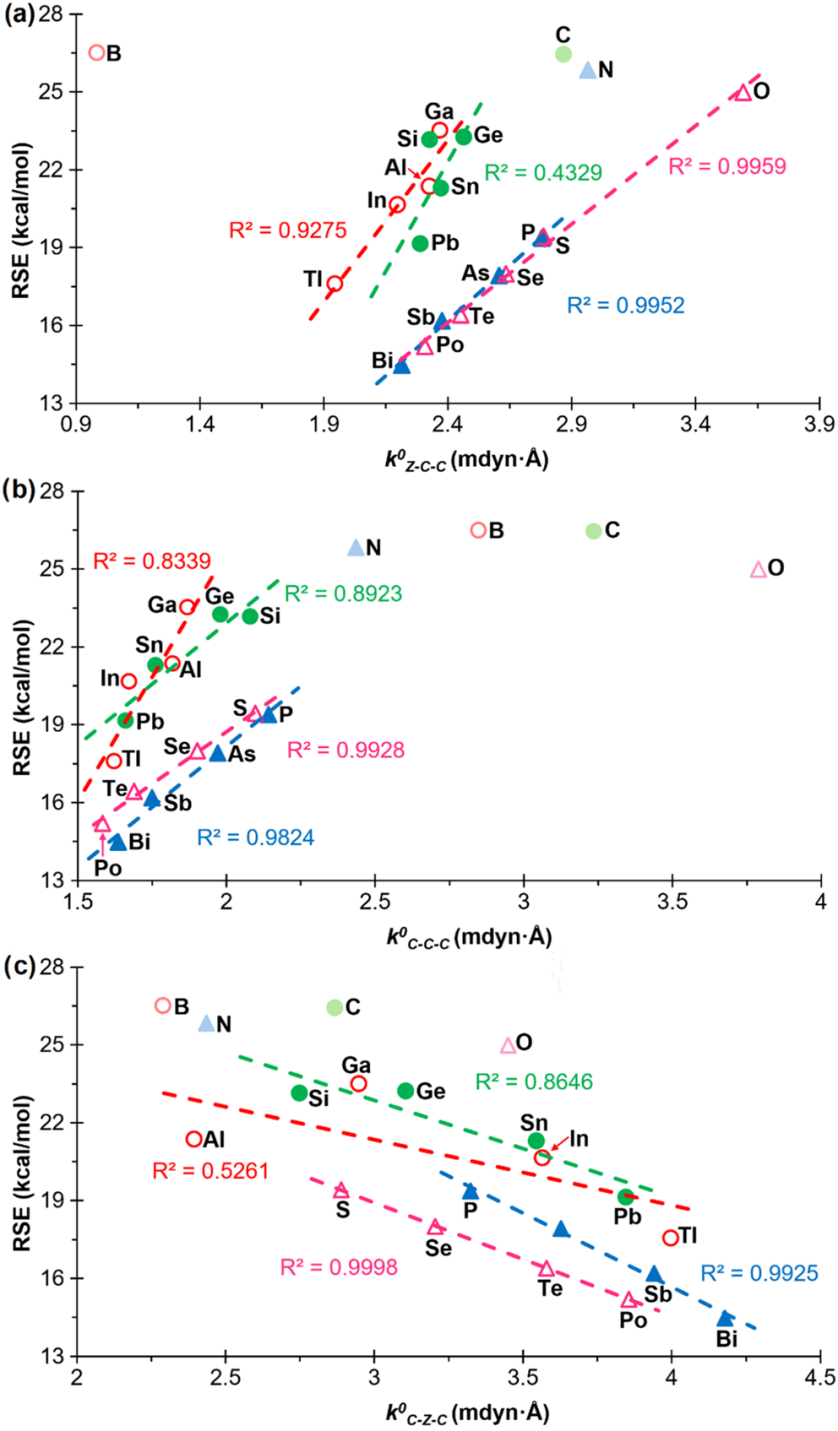
Plots of the RSEs versus the relaxed constants of the endocyclic (a) Z–C–C, (b) C–C–C and (c) C–Z–C bond angles of compounds **1** ^**El**^, for ‘El’ elements belonging to groups 13 (empty red circle), 14 (filled green circle), 15 (filled blue triangle) and 16 (empty fuchsia triangle). Compounds that have not been included in the correlations are shown with a lighter color.

In order to rationalize these findings, the relaxed force constants of the analogous Z–C–C, C–Z–C and C–C–C bond angles in the acyclic compounds **3** ^**El**^ resulting from the homodesmotic C–C bond cleavage reaction (Figure 2) were also investigated and plotted against the RSE for the corresponding 4MRs (Figure S2). Compared to the relaxed force constants in the acyclic derivatives **3** ^**El**^, the 4MRs **1** ^**El**^ demonstrate a superior linear correlation of RSE for the Z–C-C bond angle in groups 15 and 16, exhibiting a noteworthy linear correlation with positive slope. Furthermore, a correlation for the C–C–C bond angles (which is absent in the acyclic derivatives) and a reversal in the slope of the modest inverted (negative slope) linear correlations for the C–Z–C bond angles (which consistently exclude the second-row elements) were observed.

Similarly to the studies conducted on 3MRs, the potential straightforward variation of the RSEs with specific geometrical variables, such as bond angles, was also investigated in 4MRs. The best correlation with RSE was found for the C–Z–C angle that showed the expected positive slopes for all groups and even including the second-row elements (Figure 5). In the related acyclic species **3** ^**El**^, only pnictogens and chalcogens exhibited a similar direct good correlation, whereas triels and tetrels become practically invariant, as expected for approximately sp^2^ (around 120–125°) and sp^3^ (111–115°) hybridizations, respectively (Figure S2). On the contrary, the Z–C–C and C–C–C bond angles in 4MRs **1** ^**El**^ display a negatively sloped correlation only for pnictogens and chalcogens, either excluding (Z–C–C) or including (C–C–C) the second-row elements. (Figure 5). The linear correlation for the Z–C–C and C–C–C angles (Figure 5a,b) in groups 13 and 14 improves when also getting rid of the anomalous Si and Al elements (d-block contraction effect^22–24^ ).

**Figure 5:**
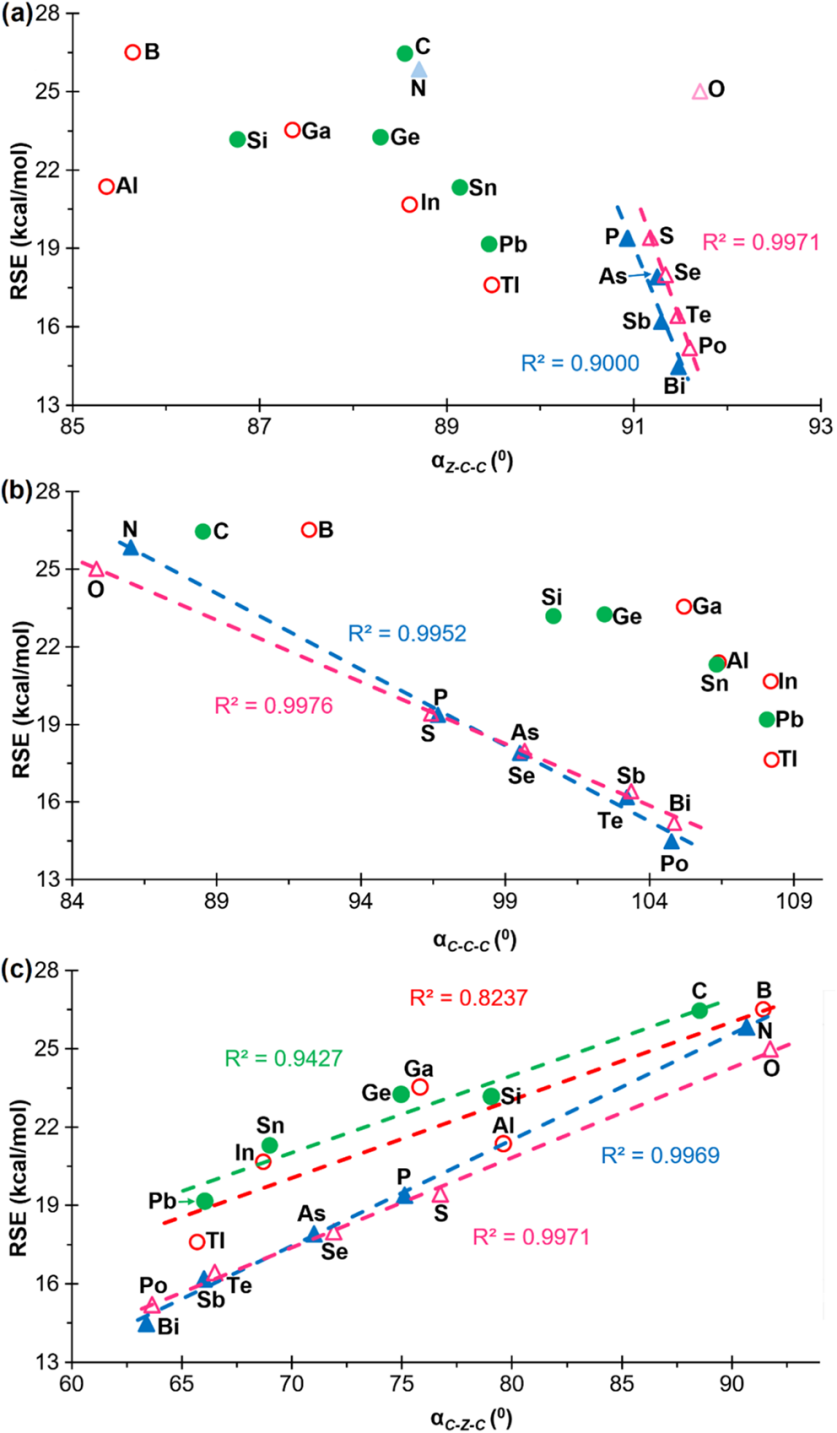
Plots of RSE versus (a) Z–C–C, (b) C–C–C and (c) C-Z-C bond angles of compounds **1** ^**El**^, for ‘El’ elements belonging to groups 13 (empty red circle), 14 (filled green circle), 15 (filled blue triangle) and 16 (empty fuchsia triangle). Compounds that have not been included in the correlations are shown with a lighter color.

#### II.2 Endocyclic bonds

The possible relationship between the RSE and the relaxed force constants of the endo- and exocyclic bonds was also investigated. First, good correlations with a positive slope were found between the RSEs and the relaxed force constants of the endocyclic Z-C bonds, practically for all groups, with *R* ^2^ = 0.9870, 0.9445, 0.9908, and 0.9948 for groups 13, 14, 15, and 16 respectively (Figure 6). Only in the case of the heterocycles containing group 13 elements, the boron and aluminum derivatives were excluded from the correlation. This behavior practically reproduces that observed for the same bond in the acyclic derivatives **3** ^**El**^ (Figure S3). From these results it can be concluded that a higher endocyclic Z–C bond strength leads to a higher RSE. In the case of the C–C bonds, which show a high dispersion for the acyclic analogues (Figure S3), the relaxed constants are almost invariant in the case of the 4MRs in groups 15 and 16, whereas relatively good correlations with a negative slope were obtained for the compounds of groups 13 and 14, excluding the Al, C and Ge derivatives from the plot. Similarly, it can be concluded that the RSEs are not related to the strength of the exocyclic Z-H bonds (Figure S3).

**Figure 6:**
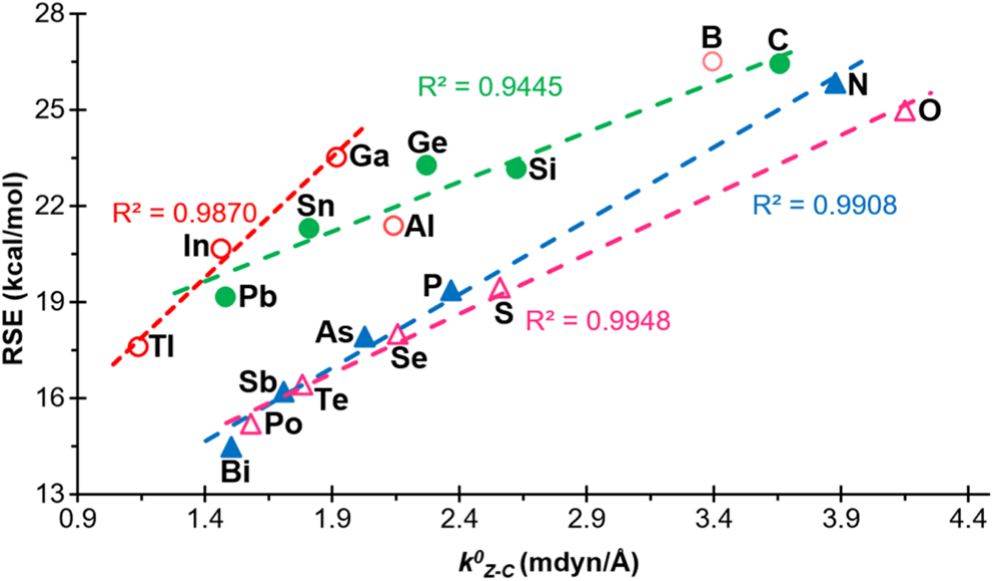
Representation of the RSEs versus the relaxed force constants of the Z-C bond of compounds **1** ^**El**^, for ‘El’ elements belonging to groups 13 (empty red circle), 14 (filled green circle), 15 (filled blue triangle) and 16 (empty fuchsia triangle). Compounds that have not been included in the correlations are shown with a lighter color.

As for the bond distances, excellent negatively sloped correlations of the RSEs with the C–Z bond distances were found (*R* ^2^ = 0.9200 and 0.9521 for groups 13 and 14, removing the second and third row elements, and *R* ^2^ = 0.9882 and 0.9854 for groups 15 and 16, respectively, in full) (Figure 7), which closely mirrors the correlation in the case of the analogous acyclic derivatives **3** ^**El**^ (Figure S6). This result suggests that a longer C–Z bond, or in other words, a heavier heteroatom, appears to be associated with a lower RSE and vice versa. In contrast, for endocyclic C–C and exocyclic Z–H bonds, no clear linear trend was found within any group in either the 4MRs (Figure S5) or the acyclic analogues (Figure S6), except for the linear behavior of the Z–H bond for group 15 in the acyclic species **3** ^**El**^ (Figure S6).

**Figure 7:**
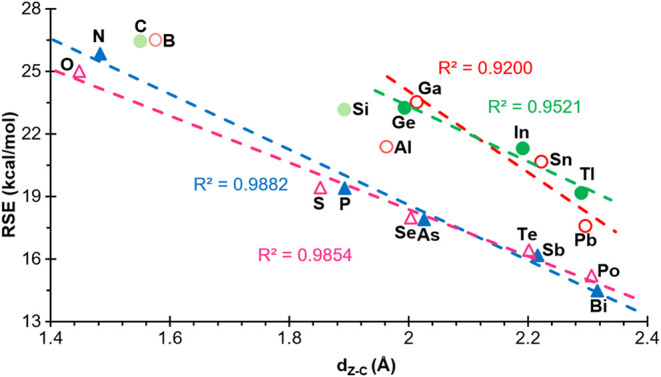
Plot of RSE versus Z–C bond distances of compounds **1** ^**El**^, for ‘El’ elements belonging to groups 13 (empty red circle), 14 (filled green circle), 15 (filled blue triangle) and 16 (empty fuchsia triangle). Compounds that have not been included in the correlations are shown with a lighter color.

### III Relationship of RSEs to Electronic Properties

#### III.1 HOMO and LUMO Energies

The relationship between the RSE and electronic properties was then investigated, including the energies of the highest occupied molecular orbital (HOMO) and lowest unoccupied molecular orbital (LUMO) of each species, as well as their corresponding HOMO–LUMO gap (Δε_H‑L_). The HOMO and LUMO energies are associated with the capacity to donate and receive electrons, respectively, and thus with the stability and reactivity of the molecule. The HOMO–LUMO gap has been employed as a descriptor of the chemical reactivity and kinetic stability of diverse compounds. Consequently, a high HOMO–LUMO gap value is indicative of a high energy LUMO and/or a low energy HOMO. This is why the addition of electrons to a high lying LUMO and the extraction of electrons from a low lying HOMO is significantly prevented, which results in a blockage in the formation of complexes^20^ and, therefore, in a reduction in reactivity (an increase in stability). It can thus be reasonably hypothesized that a cyclic molecule with high Δε_H‑L_ values would correlate with a lower RSE, which is associated with higher stability.^20^

Consequently, based on the aforementioned analysis, it was determined that the representation of the RSE in relation to the HOMO–LUMO gaps yielded satisfactory results overall, with moderately good positively sloped correlations, i.e., in the opposite direction to that previously mentioned: the RSE increases with the HOMO–LUMO gap (Figure 8). The least significant correlation, belonging to group 13, exhibited a regression coefficient *R* ^2^ = 0.8021. In all cases, moving down within each group resulted in an increase in HOMO and a decrease in LUMO energy (Figure S7), which together contributed to a reduction in the HOMO–LUMO gap.

**Figure 8:**
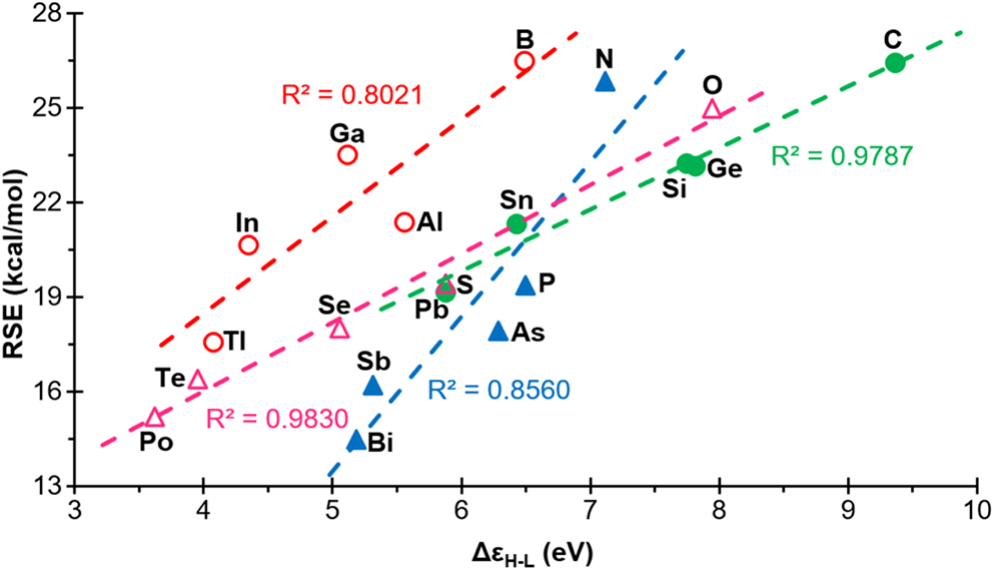
Plot of the RSEs versus the energies of the HOMO–LUMO gaps of the compounds **1** ^**El**^, for ‘El’ elements belonging to groups 13 (empty red circle), 14 (filled green circle), 15 (filled blue triangle) and 16 (empty fuchsia triangle).

#### III.2 QTAIM-Derived Parameters

The potential correlation between the RSEs and select parameters derived from Bader’s quantum theory of atoms-in-molecules (QTAIM) method^43,44^ was also examined. This approach provides a topological description of the electron density, allowing the identification of critical points and quantitative descriptors of bonding and delocalization. The first step was to analyze the Lagrangian of the kinetic energy density, *G*(*r*), at ring critical points (RCP). This magnitude has been demonstrated to correlate strongly with the RSEs of small rings.^45^ Only 4MRs **1** ^**El**^ of groups 15 and 16 exhibited a moderate linear correlation with the RSE (not shown). The results were further improved when the electron densities ρ­(r) of the RCPs were plotted, resulting in good correlations with positive slopes for groups 15 and 16 (*R* ^2^ = 0.9910 and 0.9925, respectively) (Figure 9a). Furthermore, the kinetic energy density per electron, *G*(*r*)/ρ­(*r*), did not demonstrate any acceptable improvement (not shown), despite the latter parameter having been shown to correlate well with the RSEs of some families of 3MRs.^46^ Finally, the Laplacian of the electron density, ∇^2^ρ­(*r*), exhibited good correlations for groups 15 and 16 only (Figure 9b). Typically, a negative value of ∇^2^ρ­(*r*) at RCPs is indicative of electron concentration at the ring and hence often consistent to electron delocalization or aromaticity. Conversely, positive values indicate electron depletion at the central part of the ring, thus pointing to antiaromatic or nonaromatic character. In conclusion, it was observed that only for groups 15 and 16, mainly the electron density ρ­(*r*) and ∇^2^ρ­(*r*) in the RCPs demonstrated a satisfactory linear correlation with the RSE of the corresponding 4MRs. Conversely, most of the heaviest group 13 and 14 elements tend to a roughly constant value for both AIM-derived parameters.

**Figure 9:**
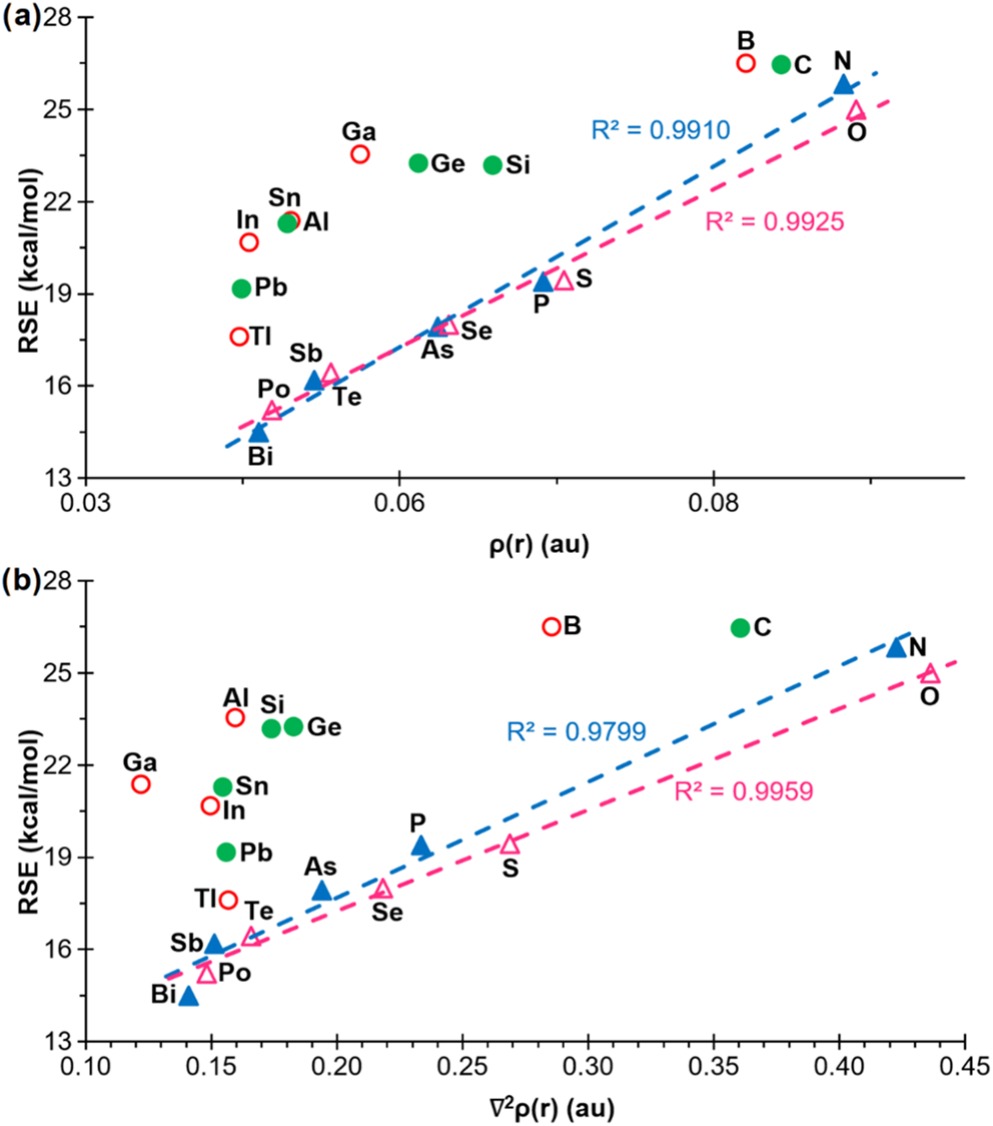
Plot of the RSEs versus (a) the electron density and (b) the Laplacian of the electron density at the RCPs in compounds **1** ^**El**^, for ‘El’ elements belonging to groups 13 (empty red circle), 14 (filled green circle), 15 (filled blue triangle) and 16 (empty fuchsia triangle).

#### III.3 p-Character of the Atomic Orbital of the Heteroatom Involved in the Formation of Endocyclic Bonds

Finally, the potential correlation between the RSEs and the p-character (in percentage) of the atomic orbital (AO) employed by the heteroatom ‘El’ for endocyclic bonds was also investigated. A notable correlation was observed for compounds belonging to groups 15 and 16 (Figure 10). This proportionality was similarly reported for the 3MRs.^20^ The same behavior has also been observed when plotting the RSE of the 4MRs **1** ^**El**^ against the p-character of the AO used by the heteroatom in the El–C bonds of the acyclic derivatives arising from homodesmotic bond cleavage in **1** ^**El**^ (Figure S8) for the same groups 15 and 16. However, as has been observed for 3MRs, in groups 13 and 14 the RSE appears to be independent of the p-character of the AO used by the heteroatom in the El-C bond, which remains essentially constant within each group and corresponds to atoms with almost invariably sp^2^ (range 64–68%p) or sp^3^ (range 72–77%p) hybridization, respectively (Figure 10). The same ranges are observed for the analogous bonds of the corresponding acyclic species **3** ^**El**^ (Figure S8).

**Figure 10:**
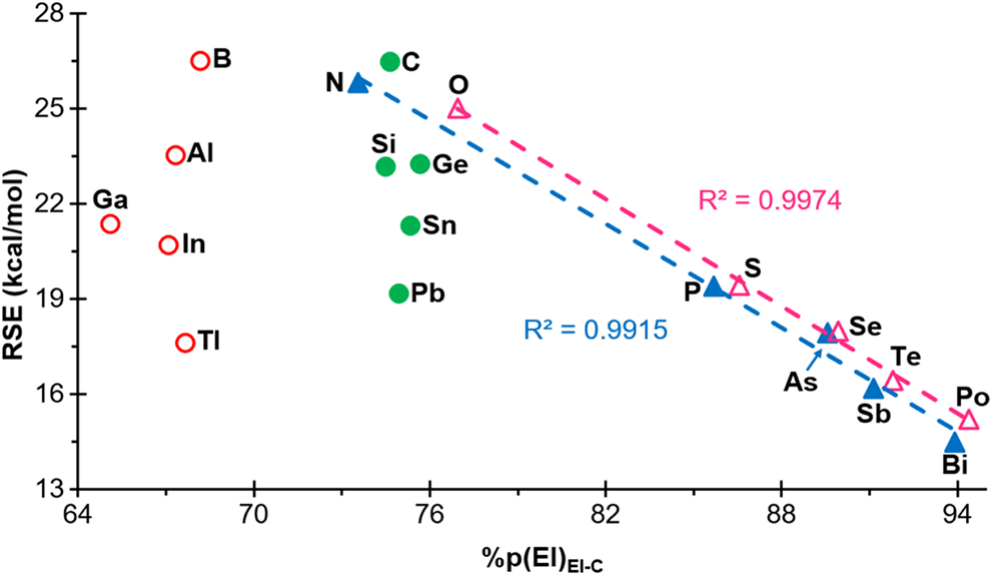
Plot of the RSEs versus the p-character of the atomic orbital used by the heteroatom in El for its endocyclic El–C bond in **1** ^**El**^ rings, for ‘El’ elements belonging to groups 13 (empty red circle), 14 (filled green circle), 15 (filled blue triangle) and 16 (empty fuchsia triangle).

The observed variation in 4MRs with heteroatoms in groups 13 and 14 indicates that the RSE is essentially affected by heteroatom size. The RSE decreases with descending in the group, presumably as a consequence of a better accommodation of geometrical strain by involving larger orbitals in heavier heteroatoms. The same strain-relieving effect, with increasing heteroatom size, is observed in groups 15 and 16. In these cases, it can coexist with the strain relaxation provided by the increase in the p-character of the AO used by the heteroatom in the endocyclic bonds, as previously reported.^20^

### IV Additive Estimation of RSEs

A fast method of estimating ring strain based on the additivity of atomic and/or endocyclic bond strain contributions was recently proposed, providing an efficient alternative for calculating RSE in 3MRs. Although atom strain contributions can furnish a rough first insight into ring strain, the most accurate method consists of summing the bond strain contributions and can now be applied to any saturated three-membered ring containing only El–El, El–Tt (where Tt = C, Si, Ge), El–Pn (where Pn = N, P, As, Sb, Bi) and El-Ch (where Ch=O, S, Se, Te, Po) bonds.^22,24,47^

The insights gained from the additive systematic method for the 3MRs have been built upon in this study, with the computational protocol being extended to 4MRs in order to assess the observed trends. In addition to the 20 RSE values calculated for the **1** ^**El**^ rings (Table 1), a set of four additional rings was included, using similar homodesmotic reactions (Figure 2) and computational level for obtaining the indicated RSEs (in kcal·mol^–1^): 1,2-oxaphosphetane (18.95), 1,2-thiaphosphetane (16.74), cyclobutanone (24.32) and 2-azetidinone (23.93). The latter enables the calculation of the contribution of CO (C=O) as a new ring atom type in this context, as well as the contributions of the new P–O, P–S, C–CO and N–CO bonds.

The initial approximation is to estimate the RSE additively (RSE^add^) using solely the atomic contributions *A*_*i*_ extended to the four constituent atoms in any given ring (eq 1). The consequence of this is the generation of a system of equations of an oversized nature, comprising 24 equations with 21 unknowns, *A*_1_^El^, the subscript ‘1’ referring to the first approximation.

1 $${{\mathrm{RSE}}_{A}}^{\mathrm{add}}=\sum_{i=1}^{4}A_{1i}$$

Following the numerical resolution of the system, a set of parameters *A*_1_^El^, (Table 2) was obtained, with a rather low Root Mean Square Error (RMSE) of 0.473 kcal·mol^–1^ and Maximum Absolute Error of 0.236 kcal·mol^–1^ (*R* ^2^ = 0.9828). This outcome is consistent with a notably linearly correlated plot of the estimated RSE_*A*_^add^ against the accurately (RC4-based) computed RSE (Figure S10). The obtained atom-strain contributions *A*_1_^El^ (Figure 11 and Table S1) are found to be in accordance with the trends observed for the 4MRs comprising a single heteroatom, as presented in this study.

**Figure 11:**
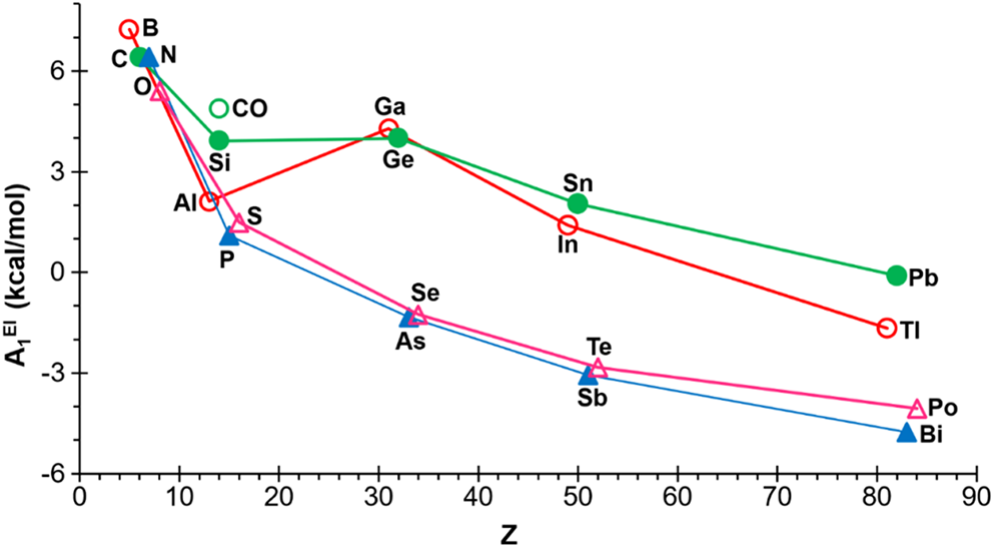
Variation of the calculated atom-strain contributions *A*_1_^El^ (kcal/mol) to RSE_*A*_^add^ with the atomic number.

**Table 2:** Calculated Bond-Strain Contributions (*B*_2_^C–El^, *B*_2_^P–El^ and *B*_2_^El‑C=O^) (kcal/mol) to RSE^add^

El	*B*_2_^C‑El^	*B*_2_^P‑El^	*B*_2_^El‑CO^
B	6.64		
Al	4.07		
Ga	5.15		
In	3.72		
Tl	2.18		
C	6.61		5.55
Si	4.97		
Ge	5.01		
Sn	4.04		
Pb	2.97		
N	6.31		5.46
P	3.08		
As	2.35		
Sb	1.49		
Bi	0.63		
O	5.89	3.37	
S	3.10	3.95	
Se	2.39		
Te	1.60		
Po	0.99		

As previously commented on the absolute RSE values (Table 1), the general trend is that as the groups go down, the atomic-strain contributions *A*_1_^El^ decrease. Again, aluminum and silicon represent inflection points in their respective series, exhibiting values that fall below the group trends. This phenomenon can be attributed to the aforementioned d-block contraction effect.

As in the case of 3MRs, replacing the atom-based parameters by bond-specific addends (*B*_2*i*_) in the additive estimation of the RSE (RSE_*B*_^add^, eq 2) should improve the methodology. In this case, solving the corresponding equidimensional system of 24 equations for the 24 bond variables *B*_2*i*_ (Table 2) results in a significant improvement, with a RMSE and MAE of 0.000 kcal/mol and an *R* ^2^ of 1.0000, highlighting the increased accuracy of this model.

2 $${{\mathrm{RSE}}_{B}}^{\mathrm{add}}=\sum_{i=1}^{4}B_{2i}$$

A small testbed comprising five new saturated 4MRs (1,3-diphosphetane, 1,3-dioxetane, 1,3-oxazetidine, 1,3-oxaphosphetane and 1,3-thiaphosphetane), which were not included in the set of rings used to estimate the contributing atom types, was used solely to evaluate the performance of the additive method. This was achieved by comparing the precise RSE values obtained from the evaluation of RC4-type homodesmotic reactions (Figure 2) with the *RSE* ^*add*^ estimation (Table 3). In contrast to the atomic strain contributions calculated for 3MRs,^22^ the RSE_*A*_^add^ approximation for 4MRs is surprisingly efficient, with a maximum deviation of only 2.50 kcal/mol when compared to the more accurate and computationally demanding RSE_RC4_ method (Table 3). In the case of the bond-based methodology, the absolute error relative to RSE_RC4_ is very similar to that of the *RSE*_*A*_^add^ method across most selected molecules. Although for the three first examples, the RSE_*A*_^add^ method yields slightly better results, for 1,3-oxaphosphethane the RSE_*B*_^add^ approach represents a significant improvement over RSE_*A*_^add^.

**Table 3:** Calculated (DLPNO–CCSDT/def2-QZVPPecp) Accurate (RC4) and Additively Estimated (add) RSEs (kcal/mol) for a Testbed of 4MRs^a^

	RSE_RC4_	RSE*_A_* ^add^	RSE_*B*_^add^
1,3-diphosphetane	13.89	15.02 (1.13)	12.33 (−1.56)
1,3-dioxetane	26.12	23.61 (−2.50)	23.57 (−2.55)
1,3-oxazetidine	26.13	24.63 (−1.51)	24.41 (−1.72)
1,3-oxaphosphetane	18.17	19.31 (1.14)	17.95 (−0.22)
1,3-thiaphosphetane	13.88	15.42 (1.54)	12.37 (−1.51)

a Signed absolute errors (kcal/mol) are in parentheses.

## Conclusion

In this work, a detailed study has been carried out of the RSEs of 4MRs containing C and only one heteroelement from groups 13–16. These heterocycles, despite being formed by four members, were found to present moderate RSEs, with values of around 25 kcal·mol^–1^ for compounds containing elements of the second period. These values decrease as one moves down each group until reaching period six, where the RSE values are between 14 and 19 kcal·mol^–1^. Undoubtedly, the most striking result is that several four-membered heterocycles (those having Bi, S, Se, Te and Po) show higher RSEs than the corresponding three-membered analogues. This apparent anomaly could arise from the relative strong σ-antiaromaticity destabilization observed for 4MRs, in comparison to the weak σ-aromaticity stabilization exhibited by the corresponding 3MRs, which contain the same heteroelements. The relationship between the calculated RSEs and some structural and electronic parameters has been studied. The strain-relieving effect with increasing heteroatom size on descending in all groups stands out and may additionally coexist with that provided by the increase of the p-character of the AO used by the heteroatom of groups 15 and 16 in the endocyclic bonds. Good correlations of the RSE with the C-Z-C bond angles (also the Z–C–C and C–C–C, especially for groups 15 and 16) and the corresponding relaxed force constants, *k* ^0^, are also observed. Similarly, good correlations are found with the Z–C bond distances and the corresponding *k*_Z‑C_^0^, which is largely related to the aforementioned effect of heteroatom size. Regarding the electronic properties, the good correlation of the RSEs with the HOMO–LUMO gaps, as well as with the electron density, ρ­(*r*), and ∇^2^ρ­(*r*) at the RCPs, is noteworthy for the four-membered heterocycles having one atom from groups 15 and 16. Finally, a rapid estimation method for ring strain is proposed, based on the additivity of atom- and endocyclic bond-strain contributions. This provides an attractive and efficient alternative approximation to RSE. The RSE_*B*_^add^ method was chosen over RSE_*A*_^add^ because it offers higher accuracy. This approach, which involves the summation of the contributions of individual bonds, can currently be applied to any saturated four-membered ring containing only C-El, P–O, P–S, C–CO, and N-CO bonds. RSE_*A*_^add^ stands out as a practical choice for these types of molecules due to its broader application (any 4MR containing any of the twenty-one studied atom types) and still good accuracy.

## Experimental Section

All compounds were optimized in redundant internal coordinates with tight convergence criteria, in gas phase and using the B3LYP functional,^48,49^ which has proven effective in similar compounds,^20^ together with the RIJCOSX^50^ algorithm and Ahlrichs’ def2-TZVP basis set functions.^51,52^ From these geometries, all electronic data were obtained through single-point calculations (SP) using a higher quality basis set including additional polarization, def2-QZVPP..^51,53^ Relativistic effects for elements in the fifth and sixth rows were considered by employing the def2-ECP effective core potentials as included in Orca (version 4.2.1)^54^ by default. In all optimizations, the Grimme correction (DFT-D4)^55^ was used, as this takes into account the majority of the contribution of dispersion forces to the energy. Energy values were corrected for the zero-point vibrational term at the optimization level and obtained by the newly developed DLPNO^56^ method for the “coupled-cluster” level with single, double, and triple perturbatively introduced excitations (CCSD­(T)).^57^ Analysis of the hybridization in the AO used for the endocyclic bonds was performed with the NBO method.^58,59^ Properties derived from the topological analysis of the electronic density were obtained with the Multiwfn program.^60^

## Supplementary Material



## Notes

Additional figures and tables; Cartesian coordinates and energies for all computed species (PDF)

## Abbreviations

4MRs: four-membered rings
RSE: ring strain energy
RC4: reaction class 4 (homodesmotic)
RCP: ring critical point
BCP: bond critical point
NBO: natural bond orbital
