# Supplementary material for: Ring Strain Energies in Four-Membered Heterocycles Containing an Element of Groups 13–16
Source: Inorg Chem. 2025 Dec 9;64(50):24808–17. doi: 10.1021/acs.inorgchem.5c04569 (PMC12728934; doi:10.1021/acs.inorgchem.5c04569)
Supplement: Supplementary file 1 [file ic5c04569_si_001.pdf]

Supporting Information

Ring strain energies in four-membered heterocycles containing an element of groups 13-16

Alicia Rey Planells,[1,2]* Antonio García Alcaraz,[1] Arturo Espinosa Ferao [1]*

[1] Departamento de Química Orgánica. Facultad de Química. Universidad de Murcia. Campus de Espinardo. 30071 Murcia, Spain. artuesp@um.es.

[2] Faculty of Pharmacy. University of Castilla-La Mancha. Calle Almansa 14 – Edif. Bioincubadora. 02008 Albacete, Spain.

Table of contents

Qualitative MO analysis of σ-aromaticity and -antiaromaticity in three- and four-membered rings S2

Figure S1. $\Delta\sigma_{ZZ}(1)_{(3MRs–4MRs)}$ vs $\Delta RSE_{(3MRs–4MRs)}$ S3

Figure S2. RSEs versus $k^0_{Z\text{-}C\text{-}C}$, $k^0_{C\text{-}C\text{-}C}$ and $k^0_{C\text{-}Z\text{-}C}$ of compounds $\mathbf{3^{El}}$ S4

Figure S3. RSEs versus $\alpha_{Z\text{-}C\text{-}C}$, $\alpha_{C\text{-}C\text{-}C}$ and $\alpha_{C\text{-}Z\text{-}C}$ of compounds $\mathbf{3^{El}}$ S5

Figure S4. RSEs versus $k^0_{Z\text{-}C}$ and $k^0_{C\text{-}C}$ of compounds $\mathbf{3^{El}}$ S6

Figure S5. RSEs versus $k^0_{C\text{-}C}$ and $k^0_{Z\text{-}H}$ of compounds $\mathbf{1^{El}}$ S7

Figure S6. RSEs versus $d_{C\text{-}C}$ and $d_{Z\text{-}H}$ of compounds $\mathbf{3^{El}}$ S8

Figure S7. RSEs versus $d_{C\text{-}C}$ and $d_{Z\text{-}H}$ of compounds $\mathbf{1^{El}}$ S9

Figure S8. RSEs versus HOMO and LUMO energies of compounds $\mathbf{1^{El}}$ S10

Figure S9. RSEs versus $\%p(El)_{El\text{-}C}$ of compounds $\mathbf{1^{El}}$ S11

Figure S10. Plot of RSE_A^{add} vs RC4-based RSE S11

Figure S11. Plot of RSE_B^{add} vs RC4-based RSE S12

Table S1. Calculated atom-strain contributions (A_1^{El}) to RSE^{add} S12

Cartesian coordinates and energies for all computed minima. S13-S45

Qualitative MO analysis of σ-aromaticity and -antiaromaticity in three- and four-membered rings.

The in-plane σ system of saturated rings can be modelled as a Hückel-like cyclic manifold of tangential hybrids. This is strongly stabilized (σ-aromatic, diatropic) for 3MRs due to the ring-centred bonding combination (symmetric, all in-phase) and no destabilizing (less bonding or just slightly anti-bonding) frontier double degeneracy in case of cyclopropane. Although there are six electrons at skeletal σ-MOs, the electron pair at the lowest σ-MO points to a marked 2-electron σ-aromaticity.

In case of cyclobutene, the σ manifold of tangential hybrids must occupy a strongly MO (all in-phase), a doubly degenerate pair with an alternating phase pattern around the ring (antibonding combination with a node through the ring centre) and one strongly antibonding MO (opposite phase on alternating sites). The σ ring system is forced to put electrons into a cyclic antibonding combination with the same qualitative phase pattern as π-antiaromatic cyclobutadiene, this giving a paratropic σ ring current in a magnetic field: that's "8-electron σ-antiaromaticity" in saturated 4MRs. The system then puckers out of planarity to partially quench that destabilising cyclic interaction (just as cyclobutadiene distorts to avoid π-antiaromaticity).

A heavy chalcogen or Bi heteroelement (El) in the ring brings in an in-plane lone pair that mixes weakly with the σ-aromatic MO in 3MRs (modest extra stabilisation) but strongly with the σ-antiaromatic MO in 4MRs (large extra destabilisation). In 3MRs, mixing nσ(El) into the lowest ring-centred bonding σ MO is stabilising if the lone pair is not too high in energy, thus effectively creating a 3-centre/4-electron σ bond spread over El–C–C. As 'El' gets heavier, the energy match improves and the σ bond system becomes more delocalised, but the pattern remains bonding around the ring. In 4MRs, when 'El' is heavy and its nσ(El) is high and diffuse, that lone pair mixes strongly into the occupied alternating MO (better energy match, better tangential overlap), resulting in a more delocalised antiaromatic σ orbital, with substantial amplitude on 'El'. This raises the energy of that MO further (electron density is being fed into an already antibonding cyclic combination). Therefore, the σ-antiaromatic penalty in those 4MRs grows faster than the σ-aromatic stabilisation in the corresponding 3MRs, so that $RSE_{4MR} > RSE_{3MR}$.

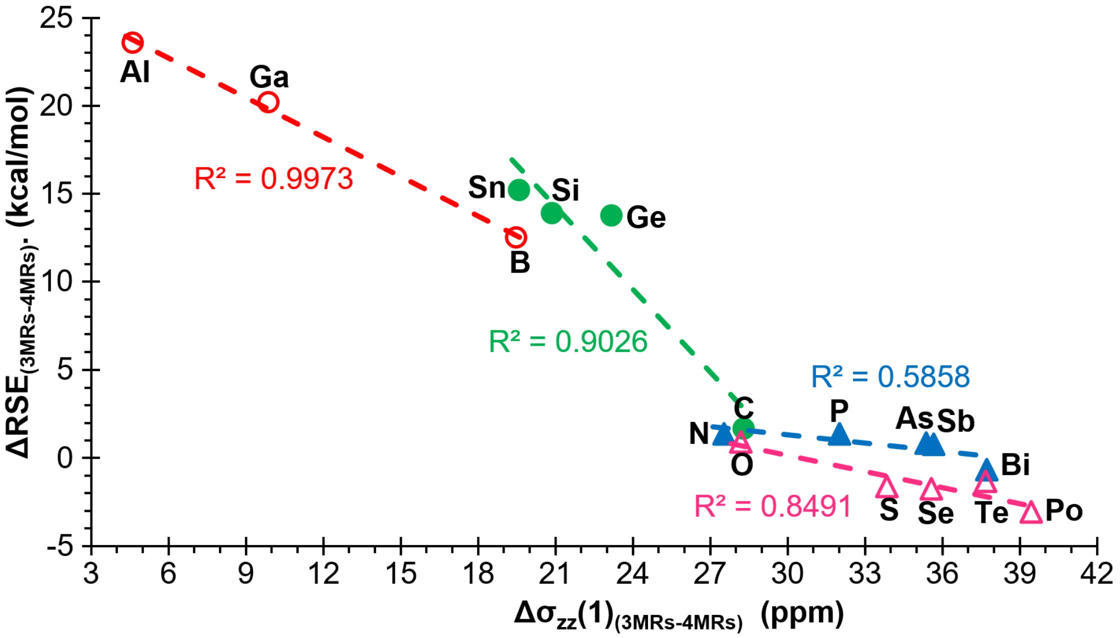

Figure S1. ΔRSE as a function of $\Delta\sigma_{ZZ}(1)$ for one heteroatom-containing 3MRs relative to 4MRs, for 'El' elements belonging to groups 13 (empty red circle), 14 (filled green circle), 15 (filled blue triangle) and 16 (empty fuchsia triangle).

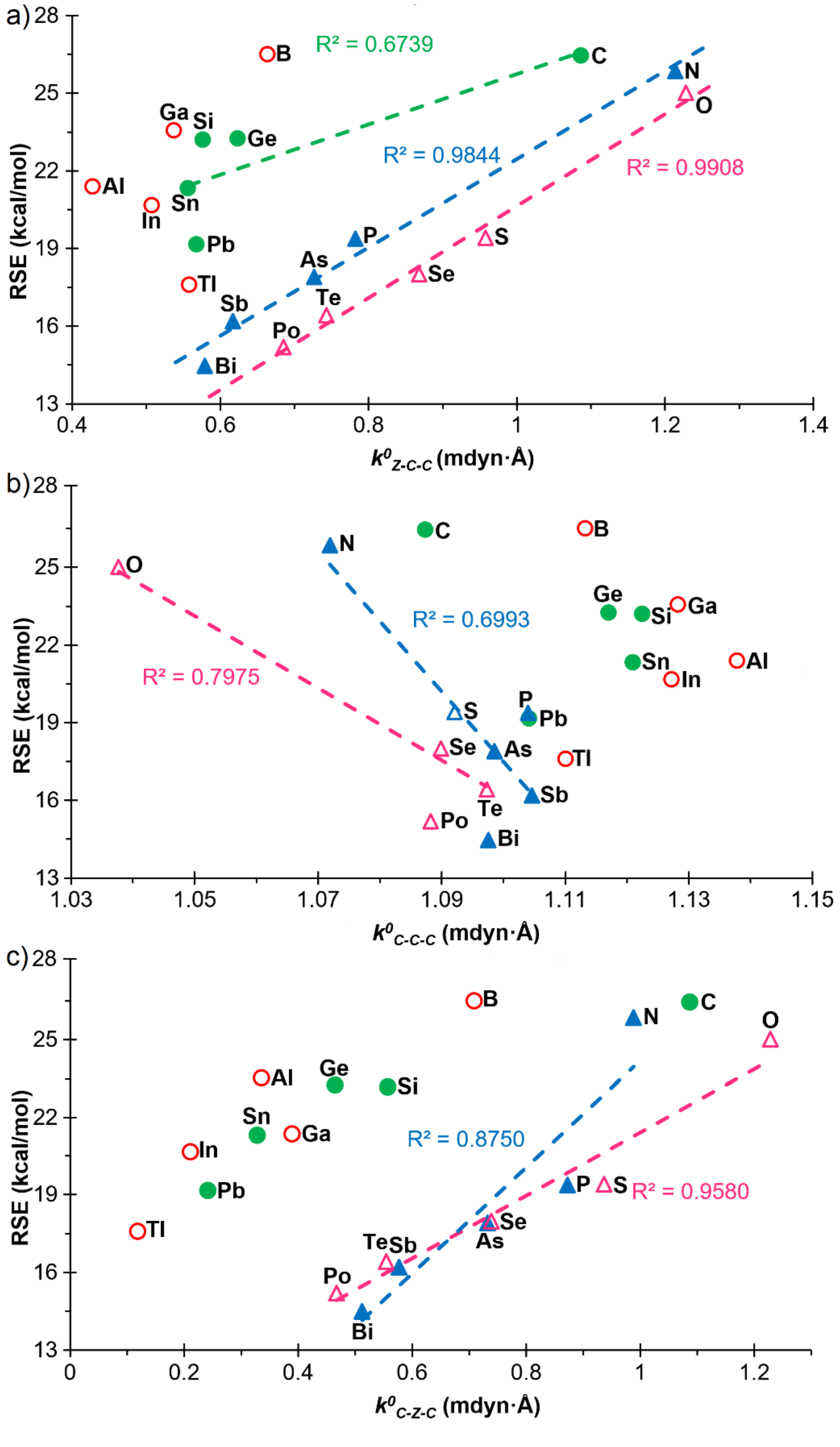

Figure S2. Plots of the RSEs versus the relaxed constants of the endocyclic (a) Z-C-C, (b) C-C-C and (c) C-Z-C bond angles of compounds $\mathbf{3}^{\mathbf{El}}$, for 'El' elements belonging to groups 13 (empty red circle), 14 (filled green circle), 15 (filled blue triangle) and 16 (empty fuchsia triangle).

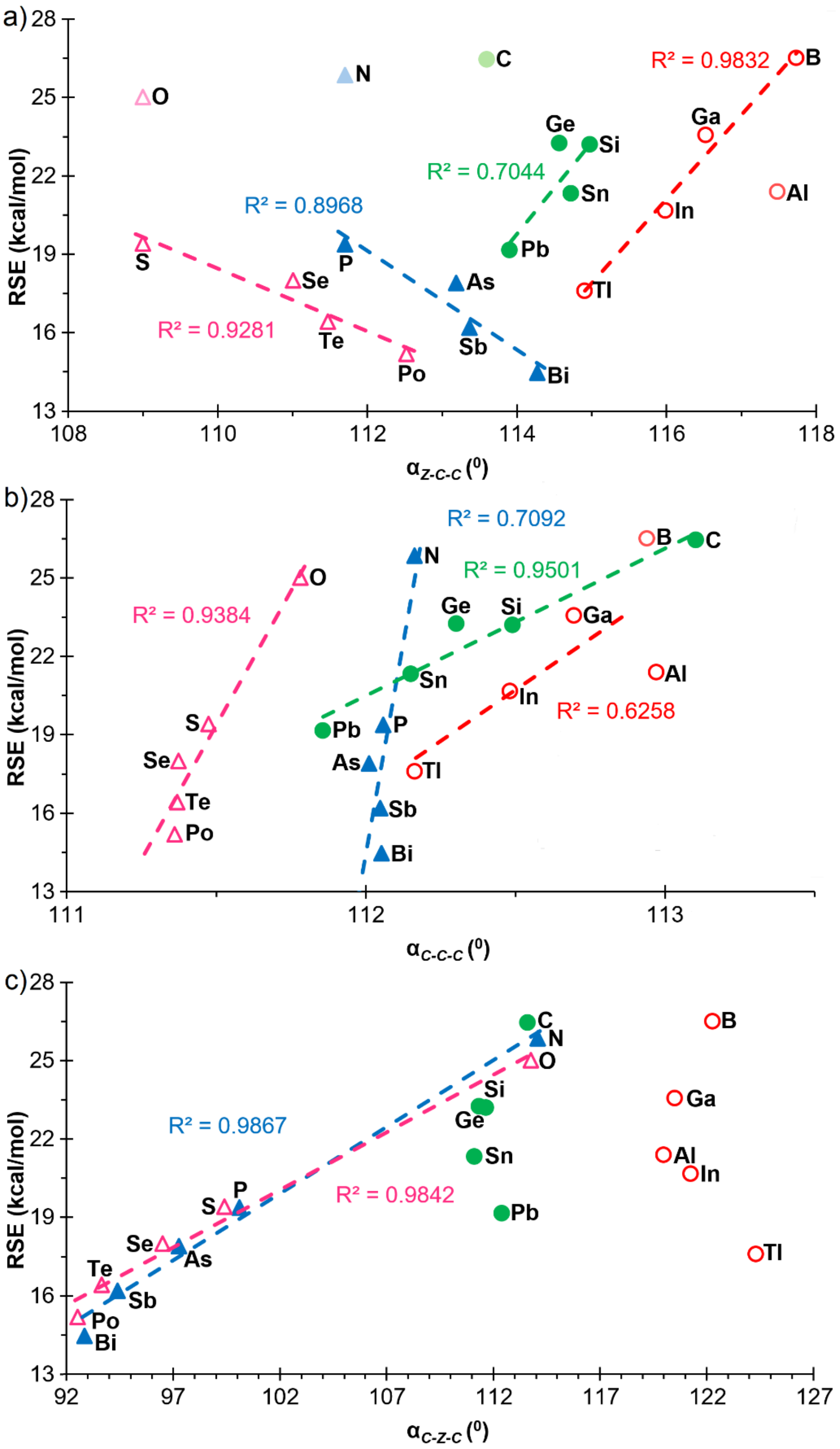

Figure S3. Plots of RSE versus (a) Z-C-C, (b) C-C-C and (c) C-Z-C bond angles of compounds $\mathbf{3}^{\mathbf{El}}$, for ‘El’ elements belonging to groups 13 (empty red circle), 14 (filled green circle), 15 (filled blue triangle) and 16 (empty fuchsia triangle). Compounds that have not been included in the correlations are shown with a lighter colour.

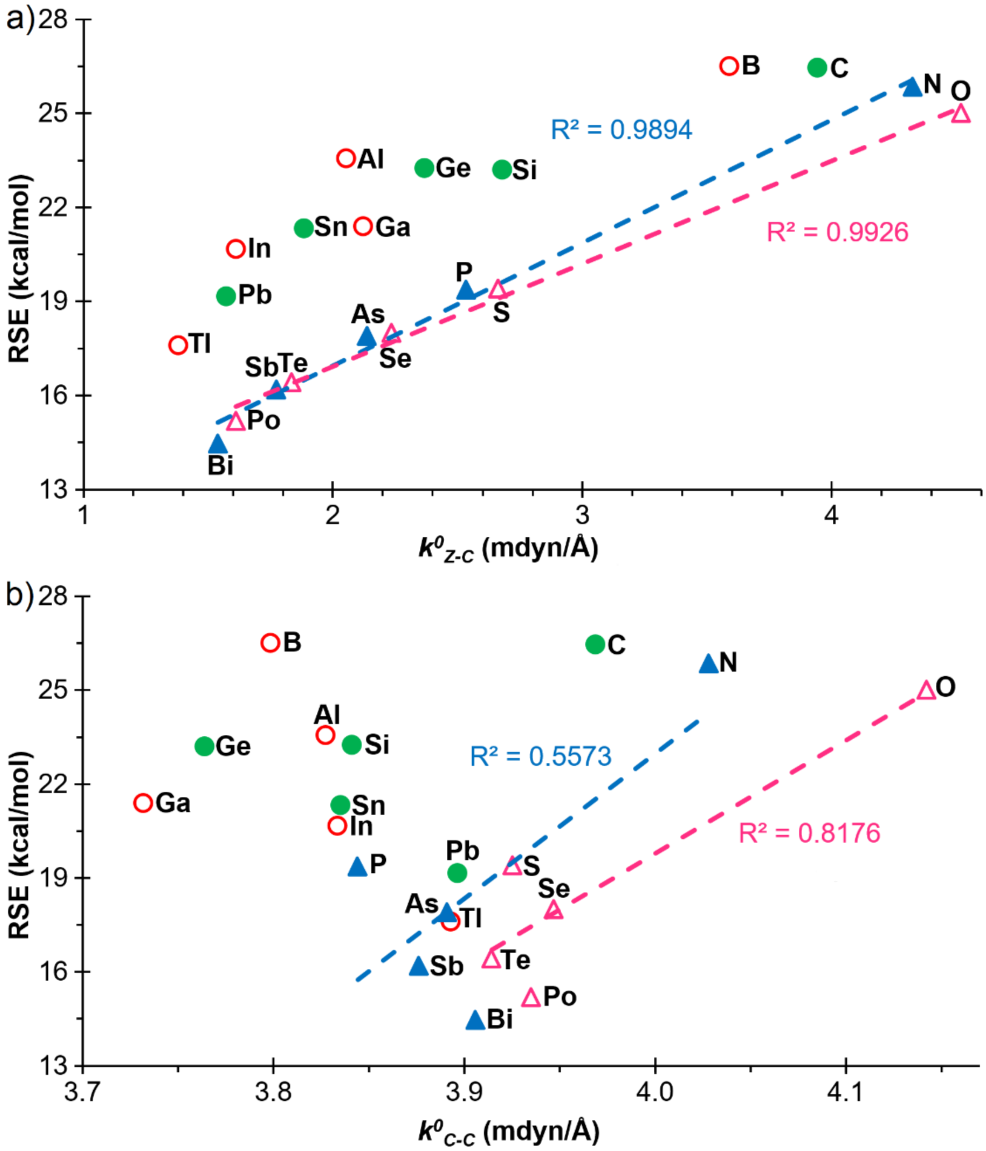

Figure S4. Plots of the RSEs versus the relaxed force constants of the (a) Z-C and (b) C-C bonds of compounds **3**$^{\mathbf{El}}$, for 'El' elements belonging to groups 13 (empty red circle), 14 (filled green circle), 15 (filled blue triangle) and 16 (empty fuchsia triangle).

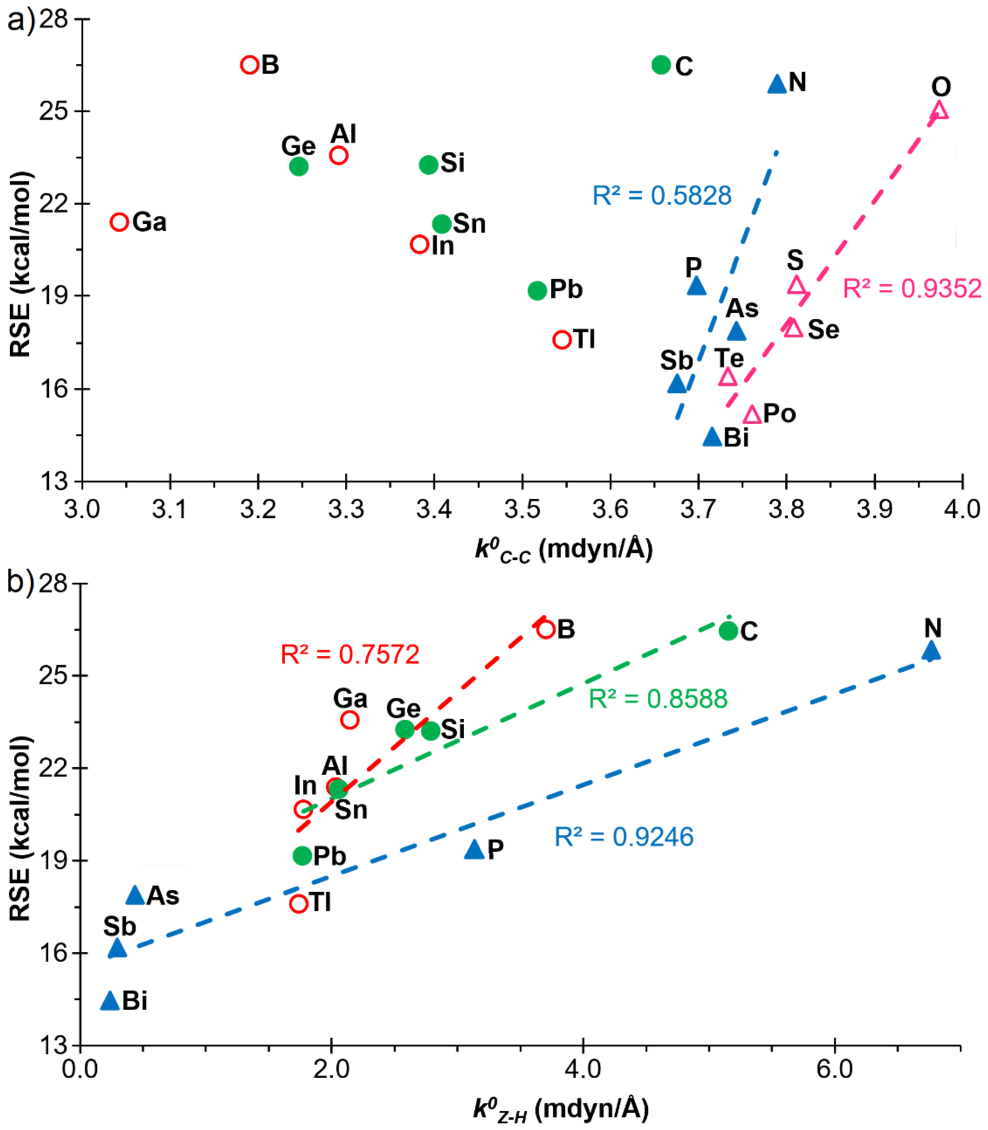

Figure S5. Plots of the RSEs versus the relaxed force constants for the (a) C-C and (b) Z-H bonds of compounds **1**$^{\mathbf{El}}$, for 'El' elements belonging to groups 13 (empty red circle), 14 (filled green circle), 15 (filled blue triangle) and 16 (empty fuchsia triangle).

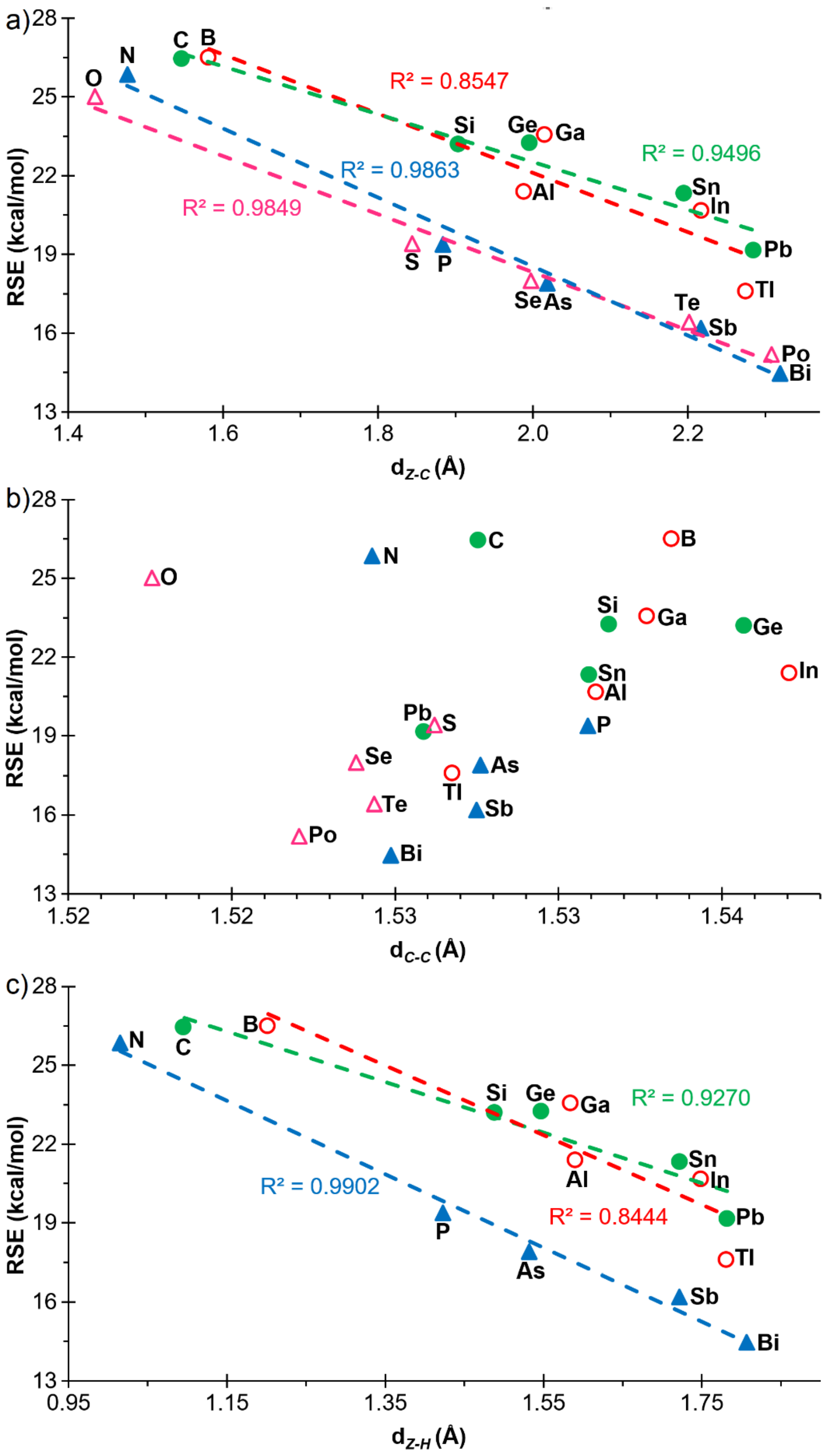

Figure S6. Plot of RSE versus (a) Z-C, (b) C-C and (c) Z-H bonds distances of compounds **3**[El], for 'El' elements belonging to groups 13 (empty red circle), 14 (filled green circle), 15 (filled blue triangle) and 16 (empty fuchsia triangle).

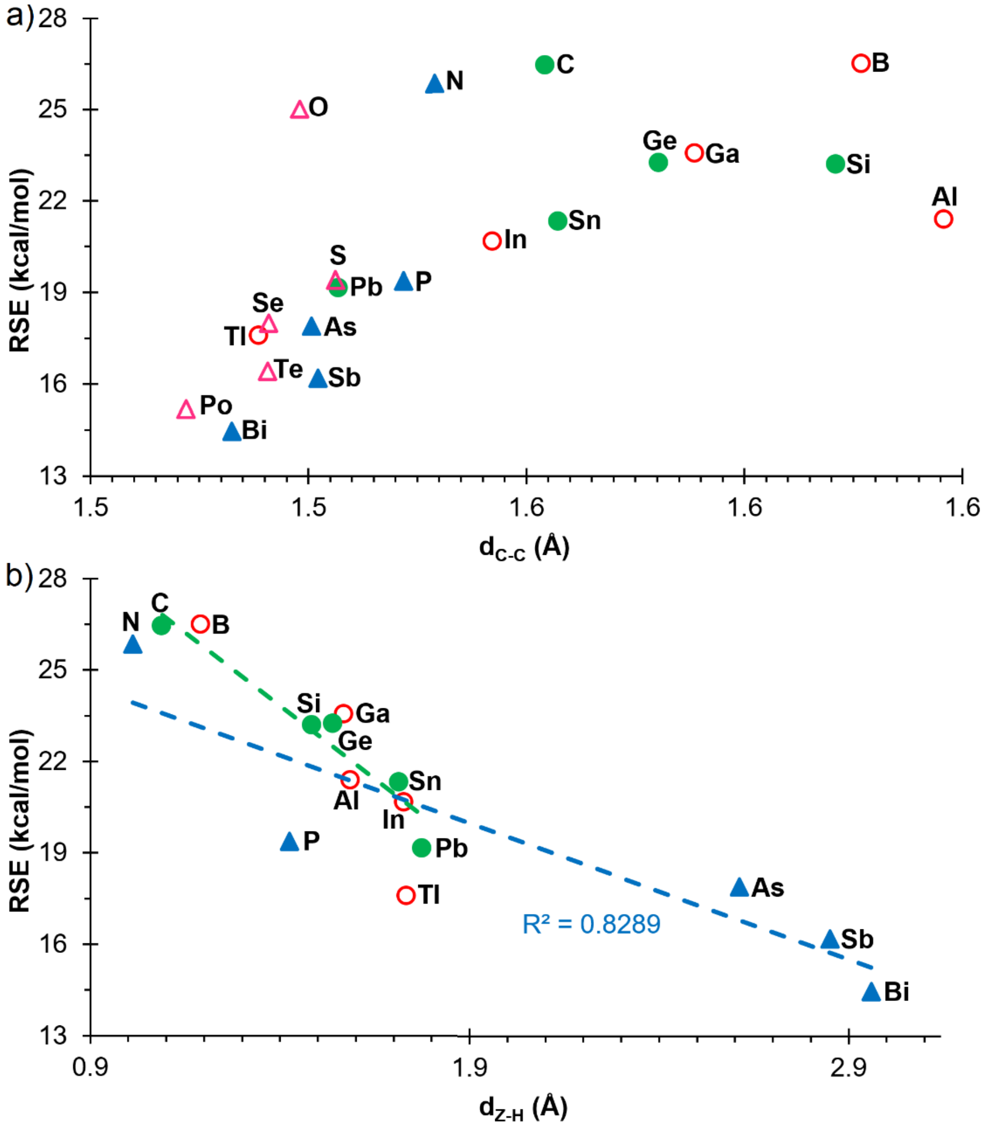

Figure S7. Plot of RSE versus (a) C-C and (b) Z-H bonds distances of compounds $\mathbf{1}^{\mathbf{El}}$, for 'El' elements belonging to groups 13 (empty red circle), 14 (filled green circle), 15 (filled blue triangle) and 16 (empty fuchsia triangle).

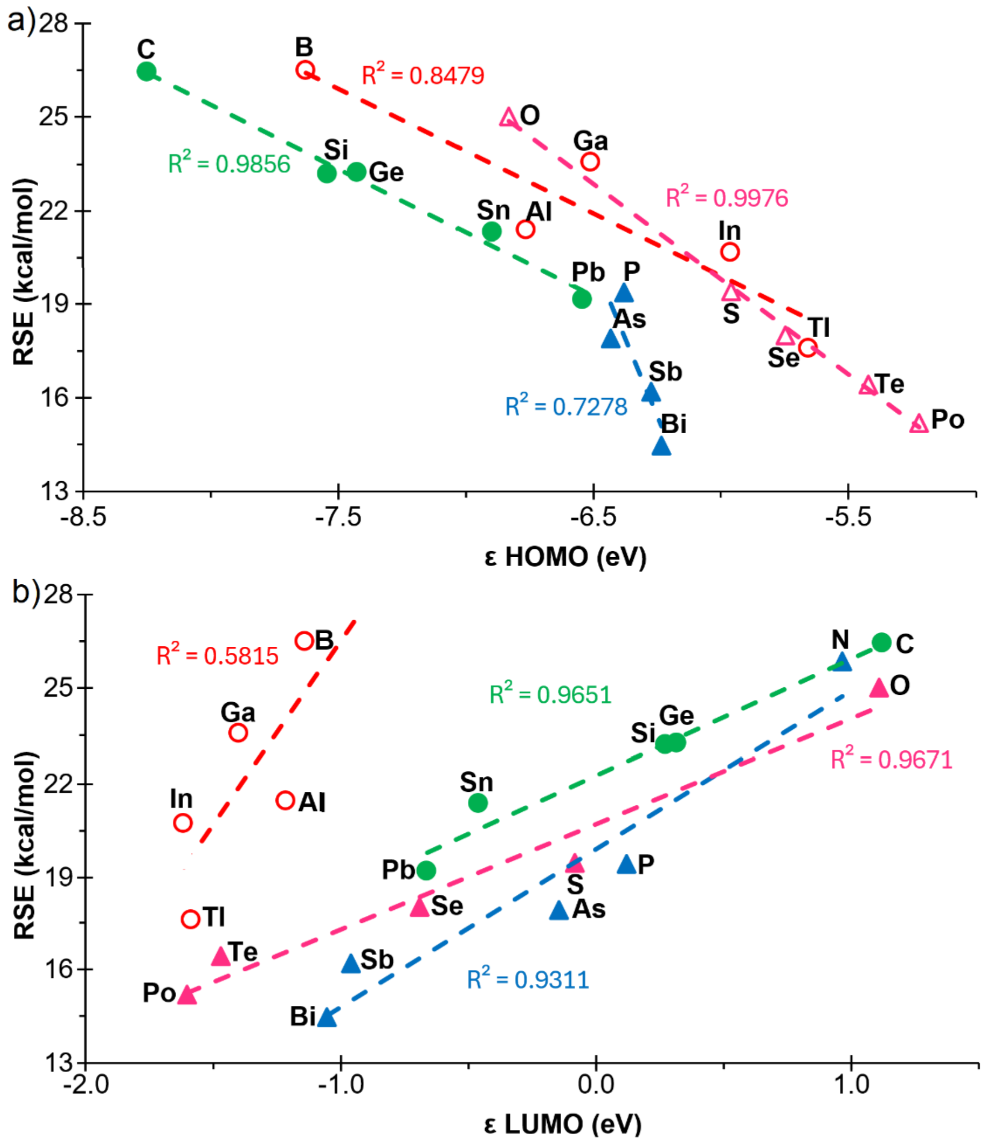

Figure S8. Plot of RSE versus (a) HOMO and (b) LUMO energies of compounds **1**$^{\mathbf{El}}$, for 'El' elements belonging to groups 13 (empty red circle), 14 (filled green circle), 15 (filled blue triangle) and 16 (empty fuchsia triangle).

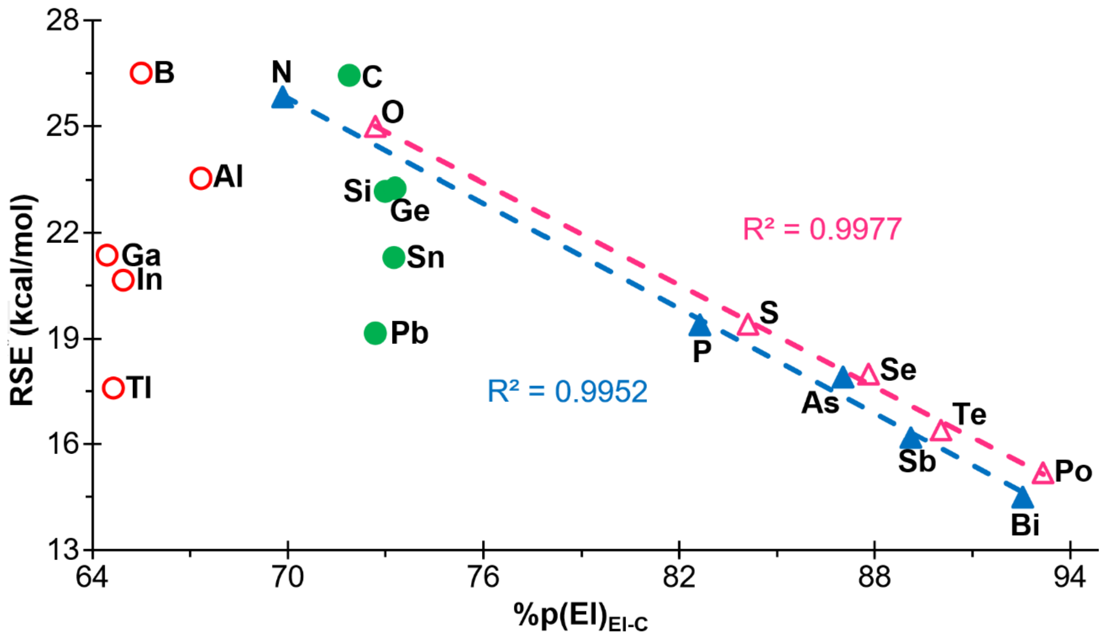

Figure S9. Plot of the RSEs versus the p-character of the atomic orbital used by the heteroatom in El for its El−C bond in **3^El^** rings, for 'El' elements belonging to groups 13 (empty red circle), 14 (filled green circle), 15 (filled blue triangle) and 16 (empty fuchsia triangle).

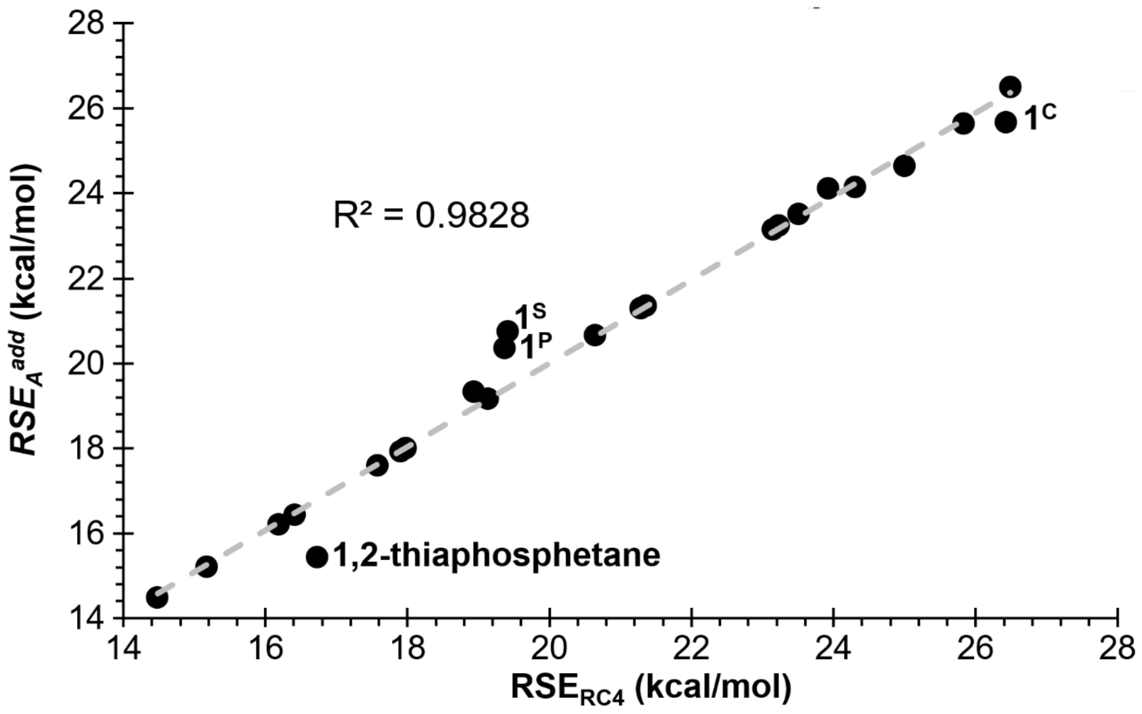

Figure S10. Plot of RSE_A^{add} against the accurately (RC4-based) computed RSE.

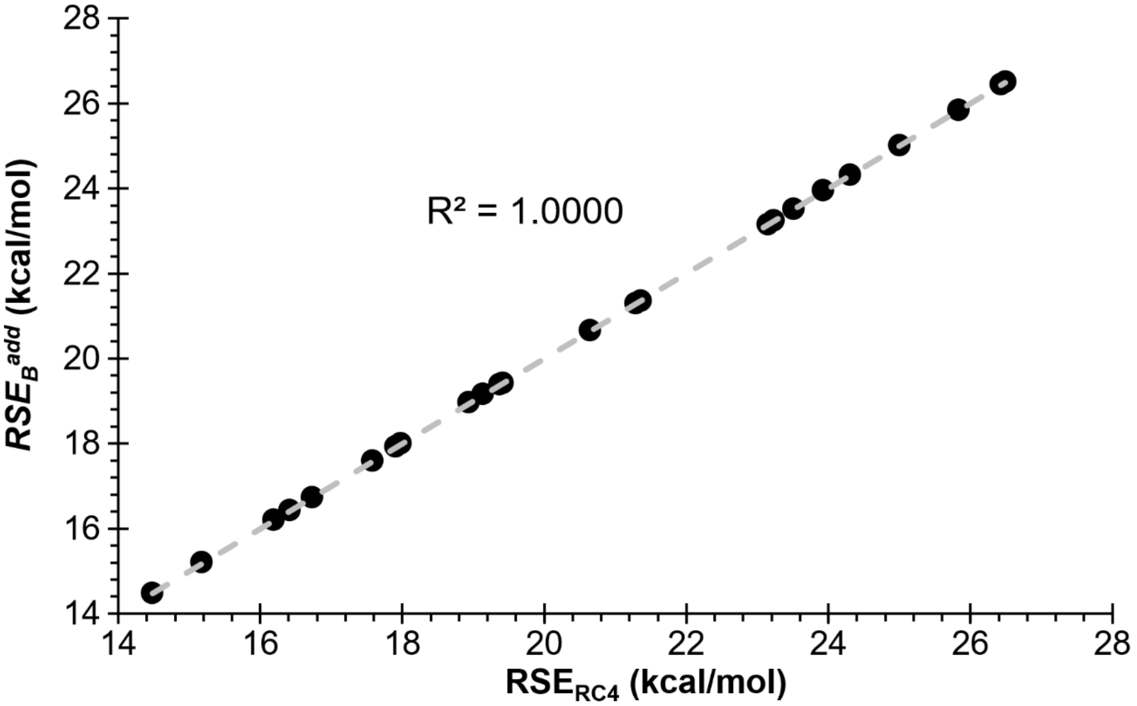

Figure S11. Plot of RSE_B^{add} against the accurately (RC4-based) computed RSE.

Table S1. Calculated atom-strain contributions A_1^{El} (kcal/mol) to RSE_A^{add}. Standard deviations are quoted in parenthesis.

El	A_1^{El}
B	7.24 (1.14)
Al	2.11 (1.14)
Ga	4.27 (1.14)
In	1.40 (1.14)
Tl	-1.67 (1.14)
C	6.42 (0.18)
Si	3.91 (1.14)
Ge	3.99 (1.14)
Sn	2.04 (1.14)
Pb	-0.10 (1.14)
N	6.40 (0.87)

El	A_1^{El}
P	1.09 (0.73)
As	-1.34 (1.14)
Sb	-3.06 (1.14)
Bi	-4.77 (1.14)
O	5.39 (0.87)
S	1.50 (0.87)
Se	-1.26 (1.14)
Te	-2.83 (1.14)
Po	-4.06 (1.14)
CO	4.88 (0.87)

Calculated structures

Cartesian coordinates (in Å), G correction (G-E) and ZPE (in hartrees) for all minima were computed at B3LYP-D4, electronic energies (in hartrees) are quoted using the default DLPNO-CCSD(T)/def2-QZVPP(ecp) level.

1^{B} E = -143.087186916 au
ZPE = 0.092346170 au

C	-0.13074221994016	-0.02877719773121	-1.12800266908124
C	-0.13060066842714	-0.02900733092634	1.12799732516763
C	0.23619879376547	0.99177897501816	0.00007825443881
H	0.48766202202244	-0.06824663613330	-2.02615260112949
H	-1.16911011988737	0.11332610101119	-1.44618473278444
H	0.48791492996115	-0.06866014258019	2.02606259162404
H	-1.16892911499942	0.11303007284834	1.44633803239663
H	1.30388140814371	1.21333667721791	0.00003453863414
H	-0.30567932773548	1.93910435280041	0.00020878222327
B	-0.01141604084475	-1.12180197520173	-0.00012152513475
H	0.28260909794156	-2.27832631632324	-0.00025800635459

1^{Al} E = -360.313861442 au
ZPE = 0.086420400 au

C	-1.28972756617169	0.35925263739813	0.07290730354437
C	0.51345709738252	2.10919326243642	0.09786849620734
C	-0.98965183501844	1.85909165012092	-0.27239148399876
H	-1.93666991475389	-0.15230558892049	-0.63944655088583
H	-1.75675458583420	0.26300110259830	1.05840374345190
H	1.05418679665420	2.75012093360611	-0.59817250697608
H	0.60944101331779	2.55961179119190	1.09108245836952
H	-1.12434643331347	2.01324675440758	-1.34563566761905
H	-1.66731813971377	2.54997047560873	0.23661730170120
Al	0.65960018762497	0.15376537264612	0.14158191125844
H	1.76628016982598	-0.98691031109374	0.15570998494694

1^{Ga} E = -2042.034054896 au
ZPE = 0.086443480 au

C	-1.29334249568700	0.39037732479698	0.08138718660034
C	0.48237162522329	2.11348053358121	0.10595055123935
C	-1.00307255889358	1.87320282404773	-0.29346409151630
H	-1.94681189839369	-0.15286835523715	-0.60069077549462
H	-1.71339394331289	0.29800106101857	1.08730494146980
H	1.05454899444245	2.75956247721311	-0.55929120762137
H	0.57277510362450	2.51682659529657	1.11885424488517
H	-1.11204288651070	2.00097363411922	-1.37330087715637
H	-1.69409150012051	2.57841952895028	0.17917202363186
H	1.79320006654679	-1.01381084221799	0.11936324982248
Ga	0.69835628308133	0.11387330843145	0.13323974413966

1^{In} E = -307.938631028 au
ZPE = 0.085330980 au

C	-1.35311227715525	0.42717646847005	0.09206489891661
C	0.44671877982835	2.17460250326902	0.11720731393527
C	-1.01393541359052	1.88432402449043	-0.30423685735311
H	-2.04920264189575	-0.08971706436028	-0.56719083203130
H	-1.73386439760608	0.35284670224178	1.11404503103266
H	0.99345222491356	2.86396755365782	-0.52482018403516
H	0.51754881715137	2.53887040421963	1.14542247974583
H	-1.09602193885816	1.98461029605389	-1.39003321613503
H	-1.71895704493586	2.60418977933133	0.12900230682798
H	2.02454429832975	-1.25076014283147	0.07402721905155
In	0.82132638381859	-0.01207242454221	0.11303684004469

1^{Tl} E = -290.190320246 au
ZPE = 0.085243650 au

C	-1.36739370620161	0.45470055989024	0.10033762585613
C	0.41929354857040	2.19042550582757	0.12528465569406
C	-1.02186836820097	1.89272441538403	-0.31864025426227
H	-2.06876710896533	-0.07817677528128	-0.53960633686119
H	-1.70910282741258	0.38265090565939	1.13507276105866
H	0.98188040750009	2.88428874987165	-0.49712006057816
H	0.48656316236610	2.51509545635907	1.16579122726599
H	-1.08535398910098	1.97387855143152	-1.40722588393996
H	-1.73505808433639	2.62104659876559	0.08936863927885
H	2.07270132392741	-1.30171627608503	0.04547380537403
Tl	0.86560243185387	-0.05687958182275	0.09978882111384

1^{C} E = -156.958825255 au
ZPE = 0.110432390 au

C	0.10384891225640	-1.14708392572185	0.15104474021719
C	1.09524277081776	0.03765016964924	0.02194715215065
C	-0.11353729825784	1.00507611444672	0.09491136985489
C	-1.03724608898954	-0.18518246901685	-0.26788016813326
H	0.27992631981293	-2.02664715893137	-0.46881726635487
H	-0.00284013267469	-1.47013734498373	1.18822336433078
H	1.56865041301702	0.05976857145157	-0.96133805608872
H	1.87105730271861	0.13651243721358	0.78169027523286
H	-0.11417255879922	1.86848208000889	-0.57106697432261
H	-0.28807538879898	1.35352387899739	1.11439174967648
H	-1.22825947300487	-0.23207295090557	-1.34147254843777
H	-1.98953391809758	-0.26761053220802	0.25680635187438

1^{Si} E = -407.992427755 au
ZPE = 0.098680510 au

C	1.21363036118763	0.12344117247515	0.00065883114573
C	-0.09971267735242	0.97144607087718	0.03151183183209
C	-1.16015490531823	-0.12047563260810	-0.32520426251003
H	1.66183292615267	0.12919408767032	-0.99527696941290

H	1.97808023909697	0.40144751784376	0.72391937949820
H	-0.09621713322957	1.83345290697991	-0.63907592548089
H	-0.27719598847533	1.34520120881003	1.04199684953810
H	-1.31914697691749	-0.17741006812715	-1.40402875668088
H	-2.12847170726026	-0.02047736289935	0.16167424149636
Si	0.11194581490986	-1.39209991931165	0.26074285268324
H	0.34758007830341	-2.63217062695496	-0.52454780316349
H	-0.04380460109724	-1.77267250475515	1.68880460105448

1^Ge^ E = -2194.809730119 au
ZPE = 0.097090590 au

C	1.22006640723062	0.16550059454406	-0.01030556515493
C	-0.09972512047951	0.98795972471829	0.01945123966915
C	-1.17163448939276	-0.08025487018075	-0.33872071008876
H	1.68673153240962	0.17391285195536	-0.99640690102081
H	1.96836699517111	0.43622906699193	0.73174020309943
H	-0.09781695653308	1.85699901206118	-0.64420568595088
H	-0.27732975496383	1.35925432986111	1.03133814268777
H	-1.35167677215081	-0.13795655259021	-1.41302225678617
H	-2.12813840644736	0.01574195125445	0.17097723551305
Ge	0.12660556902821	-1.48396829729807	0.22350137858989
H	0.37735787399521	-2.70516051266315	-0.68715842484849
H	-0.02344234786742	-1.96369968865420	1.68296864429075

1^Sn^ E = -332.744394672 au
ZPE = 0.094192730 au

C	1.24652941886925	0.22916059803025	-0.03783460459599
C	-0.09827594641703	1.00079504128892	0.00346030130490
C	-1.20169885940614	-0.02120356815621	-0.37407464824848
H	1.70497443360570	0.24228797585371	-1.02746318140779
H	1.98741068293687	0.53790559217312	0.69637373825153
H	-0.09996237588582	1.88548117964976	-0.64198144612218
H	-0.27498357855182	1.35607258976194	1.02187662051130
H	-1.37358206721180	-0.07247567081669	-1.44982801326645
H	-2.15687442393891	0.11446706464383	0.12847195499105
Sn	0.14159592404495	-1.64166377764180	0.22765024693011
H	0.42117886786752	-2.97027356874942	-0.82572034734269
H	-0.01775328591276	-2.22826752603740	1.83436932899469

1^Pb^ E = -311.149658082 au
ZPE = 0.092769880 au

C	1.25204046027899	0.26727317008891	-0.06701100803071
C	-0.09899473935717	1.00505100469492	0.00662481114367
C	-1.20694907684293	0.01656140307739	-0.40489827049594
H	1.69691645133589	0.27645110475255	-1.06159607628085
H	1.99588384224145	0.56433595442394	0.66816271278938
H	-0.10658428956567	1.91188732466729	-0.61015235134431
H	-0.27359166441647	1.32592003029359	1.03653344802660
H	-1.36382844221868	-0.03560651829047	-1.48173128857776

H	-2.16283212596273	0.14036958514005	0.09811216354039
Pb	0.15455708022767	-1.72026136339746	0.18937865329960
H	0.44629775026314	-3.06234383293846	-0.93883678947764
H	-0.00116616598350	-2.38658224251224	1.82897432540755

1[N] E = -172.984289998 au
ZPE = 0.099385470 au

C	0.08987827703764	-1.12335602707365	0.02240625671222
C	-0.12198305835359	0.97313896686972	-0.03238829228554
C	-1.13130424673646	-0.19163711469846	-0.14588506136880
H	0.46105167835707	-1.49116622497857	-0.94254675819352
H	-0.00557594770729	-1.96807856990139	0.70797829966087
H	0.17293435217101	1.35671791951864	-1.01736657137896
H	-0.38689689918024	1.81653730073569	0.60860676217568
H	-1.69936427588027	-0.27302260609684	-1.07042369158072
H	-1.81041875250412	-0.23891325800313	0.70413075296210
N	0.84975455609368	0.02712459189573	0.56568530602093
H	1.80759003670258	0.11530870173228	0.24837468727574

1[P] E = -459.240395702 au
ZPE = 0.092176040 au

C	0.06539780215962	-1.22096237302428	0.20993936514830
C	-0.16653279369422	1.07305357504153	0.14974699743768
C	-0.97195414245893	-0.17847808680339	-0.25864582971736
H	0.18335439646417	-2.11911067042568	-0.39340968886100
H	-0.08752708358879	-1.51669270638772	1.24953736875548
H	-0.22797176882019	1.94445926699418	-0.49956430082885
H	-0.38115191125952	1.38513405648915	1.17356365080192
H	-1.07487826133843	-0.21673699572038	-1.34503413954236
H	-1.97143108882077	-0.26801688053387	0.17706925225199
P	1.44025042333878	0.07564744784446	0.14263544573908
H	1.56495543801828	0.05086894652601	-1.27614499118489

1[As] E = -2353.089415369 au
ZPE = 0.090447940 au

C	0.04011942074326	-1.24515546765924	0.22456363700694
C	-0.19653506661686	1.09234322056203	0.16327141278448
C	-0.96466853870153	-0.17753555342732	-0.24487053850843
H	0.12812976652365	-2.14603238720066	-0.37823014495599
H	-0.10212568657844	-1.52513991314761	1.26894862816792
H	-0.28710967744945	1.96044463814393	-0.48547868757012
H	-0.39706337096603	1.39133415151599	1.19271786226411
H	-1.05575263641733	-0.21465116435204	-1.33274583519627
H	-1.97292531395069	-0.26841458862947	0.17285455213867
H	1.62130346742233	0.05390207865606	-1.39171578285648
As	1.55913864599110	0.08807056553834	0.14037802672517

1[Sb] E = -358.121312262 au
ZPE = 0.088399480 au

C	0.00761286247955	-1.27973368151854	0.25003976724132
C	-0.23590890266544	1.12108395917408	0.18731311206668
C	-0.95385079503870	-0.17608273679383	-0.22743679900924
H	0.04311893302748	-2.18770445952918	-0.34715079580037
H	-0.14026841631662	-1.54235879018944	1.29813116306513
H	-0.37876417799561	1.98574241919201	-0.45626956780586
H	-0.43834883496659	1.40238019581327	1.22133453100844
H	-1.02527692745583	-0.21112540545435	-1.31714264066441
H	-1.97374596716594	-0.26893945382616	0.16446829309622
H	1.73709213123542	0.06048412088107	-1.58319291878690
Sb	1.73085108486228	0.10541942225107	0.13959899558899

1^{Bi} E = -332.373412000 au
ZPE = 0.087372400 au

C	-0.01453806075559	-1.29118103579430	0.26200814254926
C	-0.26020921682974	1.12861912711434	0.19902343811948
C	-0.95233337003148	-0.17578759779370	-0.22242194235647
H	0.00750055564135	-2.20171348454125	-0.33107684413819
H	-0.15284156081671	-1.53873636199419	1.31476616856291
H	-0.41680651056041	1.99321630863097	-0.44062148951619
H	-0.45036625732954	1.39731110337969	1.23840425544414
H	-1.00907498015259	-0.20928418602703	-1.31319320018871
H	-1.98005891895151	-0.27003491970042	0.15197073793275
H	1.78351745038061	0.06249750345666	-1.66749572684841
Bi	1.81772183940560	0.11425912326923	0.13832959043943

1^{O} E = -192.845150277 au
ZPE = 0.086696970 au

C	0.09252680367263	-1.10139293716473	0.23320995896006
C	-0.11618816991554	0.96267338589706	0.17925935283564
C	-1.04307934524533	-0.18549678146335	-0.25634177358679
H	0.61194061738047	-1.66445876143633	-0.54792879415064
H	-0.15150119132852	-1.77495964347642	1.05872984820194
H	0.28440171111290	1.57717414111084	-0.63232309725369
H	-0.49389444328764	1.61542709985103	0.97040764385515
H	-1.26613818696187	-0.23622960015711	-1.32034854983142
H	-1.96909961594840	-0.26403128317623	0.31034368886936
O	0.87232919052131	0.03234280001523	0.67960903210039

1^{S} E = -515.474973390 au
ZPE = 0.084014430 au

C	0.06296749703871	-1.21677244611607	0.22842472148844
C	-0.16822916966338	1.06922425171046	0.16925079884365
C	-0.97164830958453	-0.17809384291007	-0.24494442802756
H	0.27223191752926	-2.03804218561068	-0.45380757166327
H	-0.13892824179447	-1.61344007960669	1.22346127869796
H	-0.12454110984742	1.87925398462445	-0.55587497742364
H	-0.45072396634845	1.46840024829775	1.14335498802790
H	-1.08496439997211	-0.21724579337347	-1.32841351533317

H	-1.96145122013012	-0.26637074756124	0.20730399730770
S	1.38658437277252	0.07413504054557	0.28586198808198

1^{Se} E = -2518.170000868 au
ZPE = 0.083103630 au

C	0.04237194262931	-1.24523967195601	0.22812289388913
C	-0.19431592863419	1.09288962319215	0.16734432972318
C	-0.95953955814689	-0.17684179876900	-0.23957980466176
H	0.19840053101701	-2.08817047150698	-0.43953397383448
H	-0.13199462718796	-1.59835004552471	1.24249985174392
H	-0.20646987403750	1.91443586795855	-0.54374560505105
H	-0.44066441906972	1.45575569750912	1.16309337978493
H	-1.06293754031089	-0.21500329684853	-1.32461784793233
H	-1.95783081996242	-0.26614863718853	0.19730765339325
Se	1.53427767370326	0.08772117313393	0.22372639294520

1^{Te} E = -385.306789319 au
ZPE = 0.082216980 au

C	0.02123533147192	-1.27792281637706	0.22998635992918
C	-0.22191255657710	1.12088291143526	0.16753494658806
C	-0.94367642758759	-0.17519873791160	-0.23474689946415
H	0.10306562891556	-2.14678309507835	-0.41640233616194
H	-0.13017034622812	-1.58678003807837	1.26157449092333
H	-0.31128465432377	1.95420359334519	-0.52314219784813
H	-0.43690359750195	1.44574720332384	1.18275052546050
H	-1.03443009324710	-0.21206972664720	-1.32168225288427
H	-1.95134914359066	-0.26602632120591	0.18436887505309
Te	1.72672323866880	0.10499547719420	0.14437575840431

1^{Po} E = -354.959640394 au
ZPE = 0.081711920 au

C	0.00402598544792	-1.28873477794311	0.23245921161952
C	-0.24135351905486	1.12814289565107	0.16965395695521
C	-0.94004880660407	-0.17491952820962	-0.23580310386359
H	0.06121419037942	-2.16917333764791	-0.40014723028350
H	-0.12458611509231	-1.56923434213514	1.27458209484152
H	-0.35636047885501	1.96848814578371	-0.50789410849680
H	-0.42814194263538	1.43045287924438	1.19673244914526
H	-1.02073750351862	-0.21063273234809	-1.32392973651302
H	-1.95455263558538	-0.26680849138780	0.17026348961111
Po	1.82183821551830	0.11346773899252	0.09870025698429

2^{B} E = -208.948502064 au
ZPE = 0.151887840 au

C	-3.86318702547389	-1.23738808192765	-0.05836960388143
H	-3.49707521339221	-2.20929683425492	0.27640587757937
H	-3.66016847087671	-1.17886657831823	-1.13977460299086
H	-4.95211831889557	-1.20854737854273	0.02709166117868
C	-1.76877265372226	-0.00493893856511	1.28270127944968

H	-1.13696194077257	0.73918719450207	0.77437132859107
H	-1.91322228023880	0.44038310178441	2.27948393316865
C	-1.01477905079229	-1.33234145878824	1.40943724394390
H	-0.86052940194579	-1.75747812859723	0.41261211051968
H	-1.63827341973272	-2.05119861830311	1.95020338816043
C	0.35529902549258	-1.18553984047262	2.13146160739781
H	0.95979294509865	-0.46944517197513	1.56661013090551
H	0.16986905258229	-0.76143040175487	3.12296502256123
B	-3.16368302827579	0.00551761989912	0.57450558142607
H	-3.70951955572587	1.07028233644708	0.48033853231864
B	0.98689890652640	-2.60664537410679	2.18602086629684
H	0.74459093933274	-3.35146276185150	3.08935015322149
H	1.65504635081182	-3.01760093517453	1.28373418015323

2^{Al} E = -643.391692050 au
ZPE = 0.136293490 au

C	-4.30328103924912	-1.39278568271124	-0.08592620326254
H	-3.95438693377428	-2.30204183069163	0.40959148917285
H	-4.11947155861944	-1.52802204028113	-1.15888485376153
H	-5.38782619740394	-1.32907984514813	0.03526038670610
C	-1.58371328669856	0.13176710671489	1.32939741645143
H	-0.92710889234814	0.79609791806382	0.74963646804263
H	-1.62184545982003	0.59980545937461	2.32347050813231
C	-0.94321459967564	-1.26091885679071	1.43815169124093
H	-0.90028593591290	-1.71959188455586	0.44367809296713
H	-1.59971248952524	-1.91869220404467	2.01900676582722
C	0.46208078288219	-1.27084037891379	2.06210842824765
H	1.12569039872801	-0.60547184598917	1.49162721750717
H	0.42735113030197	-0.81412766652119	3.06166041013606
Al	-3.38531862187578	0.21912573007566	0.54494067880284
H	-4.09306264922585	1.63508233071009	0.39598525742763
Al	1.34927493421161	-3.01383044407081	2.22709690829362
H	2.81146935600372	-3.11138503330560	2.83401579520930
H	0.62015768200144	-4.34383009191510	1.75970646285919

2^{Ga} E = -4006.839288139 au
ZPE = 0.136511480 au

C	-4.31618005863422	-1.41572453051138	-0.09568843019773
H	-3.73670354801823	-2.32364968677319	0.07116610914529
H	-4.56497554117141	-1.34961112183493	-1.15800339734278
H	-5.26253240624257	-1.50156903283598	0.44508811058838
C	-1.55097915734542	0.14505053288798	1.31773024872310
H	-0.89292486429998	0.82159361282126	0.75947798601057
H	-1.63571300123748	0.59569045356575	2.31461863636745
C	-0.92167354458266	-1.24826807203136	1.41870396850894
H	-0.83242969960065	-1.68273230714865	0.41702845076746
H	-1.59911098094626	-1.91878287470757	1.95851355832076
C	0.45079010438987	-1.25705701825433	2.10137675279846
H	1.14428885600109	-0.58998688873559	1.57459935718161

H	0.37623707488094	-0.84017363046930	3.11323615092162
Ga	-3.35798496059337	0.22385073106298	0.48192841243172
H	-4.06116101721417	1.62757089304565	0.28142385941646
Ga	1.33907586128436	-3.02793799589106	2.25295434786576
H	0.64712084171803	-4.32768177695809	1.68337021296788
H	2.75165265161223	-3.15932053723206	2.94299857552497

2^{In} E = -538.643016478 au
ZPE = 0.133253730 au

C	-4.46082162044806	-1.50195790736998	-0.16081443527910
H	-3.86122694303814	-2.38939237868666	0.03739243967089
H	-4.66738279399755	-1.44455949429652	-1.23111286356231
H	-5.41741110856666	-1.59464983129674	0.35657090275977
C	-1.45238882953143	0.18869924357340	1.40064693717697
H	-0.79211744805140	0.86080892737599	0.84334347019776
H	-1.53014590711602	0.61631072616366	2.40586080430963
C	-0.86074499026952	-1.22232401454049	1.46168723290303
H	-0.79565975506615	-1.63824179218479	0.45033560216628
H	-1.54694353205131	-1.88673775518930	1.99801229786163
C	0.51945798054033	-1.26891131518016	2.12480133988151
H	1.22526940549083	-0.62249039202475	1.59315233326461
H	0.47024736398151	-0.87560056618687	3.14541417592326
In	-3.44385650758049	0.31428533871518	0.49265122988769
H	-4.23447239272089	1.85731248216286	0.29215224953864
In	1.43192247380401	-3.25239300542359	2.24573274933168
H	2.98885264779947	-3.48603329681001	2.98611741377490
H	0.59609164682150	-4.63431087880120	1.59390431019313

2^{Tl} E = -503.137775223 au
ZPE = 0.132672010 au

C	-4.43544280855651	-1.53509622358540	-0.18913203410650
H	-3.81414552468144	-2.39429044754977	0.05329057938720
H	-4.57739290031562	-1.47483014324397	-1.26809815707435
H	-5.41074202976218	-1.63323464771988	0.28643653746217
C	-1.42207587985655	0.21685112099595	1.44209078339112
H	-0.77415475131750	0.88693168175811	0.87173417666507
H	-1.50673413245367	0.63962725249600	2.44632616358140
C	-0.86054425842847	-1.20253161738335	1.48196760481867
H	-0.81589501062407	-1.61293130509313	0.46812779252202
H	-1.54671613058839	-1.85685150650912	2.02909356834484
C	0.52525346098510	-1.26092819504660	2.12192056696358
H	1.24435921198087	-0.64624578433714	1.57518143729039
H	0.50680085219968	-0.88525159899479	3.14799461854240
Tl	-3.46725243252569	0.35749222972856	0.51724440458016
H	-4.31787357955183	1.89389360501700	0.33667171737430
Tl	1.40796424675543	-3.31732015847098	2.22809813208493
H	2.99370895922024	-3.63094326030471	2.92195079369702
H	0.47525109752067	-4.67587275175674	1.59401110447552

2^{C} E = -236.703354635 au
ZPE = 0.188284380 au

C	-4.81123793989231	-0.01855235276526	-0.02139866622760
H	-4.39224682074543	-1.03015460186581	-0.05314975166641
H	-4.46123063108326	0.48304281290738	-0.93023510794980
C	-4.25943283370719	0.71400094532152	1.19988790242217
H	-4.67679006863410	1.72748531437961	1.23113058168455
H	-4.61169392833130	0.21401692855225	2.11001753927385
C	-2.18332162790775	1.52760602779906	2.44768417179077
H	-2.53331961428678	1.02599972294253	3.35651801128821
H	-2.60231824784880	2.53920557967756	2.47944803513406
C	-0.65874949073528	1.60562602899937	2.46300355051845
H	-0.28252990569421	2.13031655606763	1.58094380728155
H	-0.29317078740689	2.13478745435738	3.34542989884634
H	-0.21369525470195	0.60709711611855	2.46611526440475
C	-6.33581032006162	-0.09656382467571	-0.03672840990881
H	-6.78085895633773	0.90196748550834	-0.03985296639261
H	-6.70138563641048	-0.62573118127961	-0.91915262733146
H	-6.71203969527325	-0.62124431903615	0.84533312838348
C	-2.73512987795606	0.79506177182357	1.22639400600541
H	-2.38287023043094	1.29505236813313	0.31626747171273
H	-2.31777233255463	-0.21842223296536	1.19514340073035

2^{Si} E = -738.751385010 au
ZPE = 0.162069430 au

C	-1.67373916570367	-0.04106802501774	1.22577052037987
H	-1.12895073016302	0.35629645729363	0.36153158439454
H	-1.50446953038569	0.67224850414129	2.04077052703693
C	-1.10178594667231	-1.41177936034051	1.61237460288408
H	-1.26127323013491	-2.11645488453507	0.78948868033753
H	-1.66472777262251	-1.81364636526031	2.46161169092633
C	0.39188082336252	-1.37305815773598	1.96712489090094
H	0.96639576590409	-0.98762842858983	1.11767515897897
H	0.55955924760729	-0.66814828521660	2.78882741515907
C	-4.12561644412383	1.65552495432535	0.33595952221361
H	-5.19319830490423	1.63994938056269	0.10690522100285
H	-3.96760924455116	2.37240610469720	1.14490900769137
H	-3.59929808443025	2.02470241025763	-0.54730452300241
Si	1.10280457786334	-3.04379555447812	2.46459246517306
H	2.54683054418269	-2.92979751015036	2.78901892704553
H	0.39213805199654	-3.57178174092646	3.65697834098167
H	0.94506843331737	-4.02449246098998	1.36074889262711
Si	-3.51324514618395	-0.05229687491755	0.82023015376644
H	-4.27023860177651	-0.54026586268623	2.00422325574384
H	-3.76374716258179	-1.00932492043304	-0.29068138424134

2^{Ge} E = -4312.388491410 au
ZPE = 0.158578850 au

C	-1.63807356477080	-0.04687557743701	1.24088268632152

H	-1.10317952827505	0.35404727089013	0.37480261050975
H	-1.48957496947778	0.66636656029795	2.05709218733140
C	-1.07142248415344	-1.41627778954591	1.62573728170807
H	-1.22994206187927	-2.12058927673434	0.80240063350950
H	-1.63384895864298	-1.81786742959459	2.47539505774622
C	0.41865362605998	-1.37049992410597	1.97794615579398
H	0.99874747141393	-0.99474958472836	1.13073506059581
H	0.59188625418815	-0.68015423521187	2.80793815062910
C	-4.19289069221147	1.72596755946613	0.30287047074077
H	-5.25871222024296	1.71049089294897	0.07523845158321
H	-4.02337604749148	2.42711475198112	1.12066234738956
H	-3.65553502885668	2.08224161205624	-0.57664502942529
Ge	-3.56480040369804	-0.07009577865478	0.80550552518810
H	-4.35707187955156	-0.57616031421124	2.03349674320944
H	-3.81437428544684	-1.05804123399709	-0.35768615139093
Ge	1.15773498304045	-3.12744406701674	2.49080964704171
H	2.66448826362742	-3.02268485693509	2.79890375066240
H	0.43310064792420	-3.66007346516489	3.74412047443759
H	0.95688681844422	-4.14184279430264	1.34638591641808

2^{Sn} E = -588.253769328 au
ZPE = 0.151794280 au

C	-1.56730456340886	-0.07212354574919	1.30638192967813
H	-1.03792278133551	0.34964890182119	0.44813986047255
H	-1.42940734197625	0.62378829627971	2.13785073808825
C	-1.00400300378204	-1.45084152856182	1.66070025457987
H	-1.15816879483580	-2.13533096499064	0.82002144702606
H	-1.56946248258381	-1.87389430040618	2.49785281268793
C	0.48377038117994	-1.40709336979774	2.02080536241949
H	1.07115161639211	-1.01122954448728	1.18907028219816
H	0.65449402261585	-0.74780984341801	2.87498797294466
C	-4.33667524849649	1.87352626048813	0.18827320230177
H	-5.40509906138436	1.86601912638004	-0.02317776954650
H	-4.13687331828579	2.60156486378389	0.97384951303107
H	-3.79942390866033	2.16841799890037	-0.71256218643225
Sn	-3.68556846917801	-0.08843753303807	0.82204957404170
H	-4.57862235095854	-0.57999514989960	2.20856459002098
H	-3.97050487973663	-1.23052426308013	-0.43336959614649
Sn	1.30163720464227	-3.35182675755484	2.53033249918620
H	1.09241961779510	-4.43266227243599	1.21376528207815
H	2.97393405373863	-3.24046913877056	2.89577233054399
H	0.47559766825852	-3.98721765546328	3.89399745082626

2^{Pb} E = -545.058407315 au
ZPE = 0.148704360 au

C	-1.49873094057553	-0.09693473864868	1.29215749704898
H	-0.98718919568365	0.30666646378204	0.41699425008604
H	-1.37746606075823	0.61377561146483	2.11107419364147
C	-0.96324669242986	-1.47687039593228	1.66610260022792

H	-1.12568130520850	-2.17241737100114	0.83679381264397
H	-1.53206693154895	-1.87668925593737	2.51149558723503
C	0.52115875328829	-1.43526818614635	2.02063327392331
H	1.12539453759232	-1.08564438129841	1.18273710534981
H	0.71231896044085	-0.78171134811172	2.87248075268793
C	-4.41669098706949	1.93207123740414	0.19873517495648
H	-5.48514700823671	1.89258991094493	-0.00039345716005
H	-4.21549151539229	2.62355569826845	1.01399955947466
H	-3.88255495451219	2.24515182499418	-0.69573132517278
Pb	-3.70413488677172	-0.11663698193817	0.79175500262555
H	-4.61616261667997	-0.68058531076062	2.21150536709928
H	-3.99023063463720	-1.26094591956856	-0.54020605014812
Pb	1.33280495596199	-3.46325890575436	2.58736348432521
H	1.09673475007095	-4.59788210542396	1.24694818436163
H	3.05961825520704	-3.38549885670453	2.97131824417222
H	0.46048872694286	-4.08045690963240	4.00083612262142

2^N^ E = -268.757221890 au
ZPE = 0.166530910 au

C	-1.78567835555186	0.14155708318376	1.22590584804848
H	-1.37234663478210	0.47170409950260	0.25489883364102
H	-1.43536666004239	0.86679988954343	1.96887365188186
C	-1.24592230558288	-1.24166612565571	1.56169339593036
H	-1.59946716449139	-1.97037593201924	0.82499328197073
H	-1.65120617060356	-1.55113449297925	2.53071782944088
C	0.27582698217135	-1.29276076457662	1.59885569574774
H	0.67418724720956	-0.99369834220326	0.62476413571861
H	0.64192739507636	-0.55393536803339	2.32892908756141
C	-3.80484162999189	1.44629192131281	0.85852492659062
H	-4.89326549161620	1.37773432888738	0.82947520463031
H	-3.53927007453038	2.19733248326230	1.60690571102236
H	-3.45486535799754	1.81600035872599	-0.11992227567267
N	-3.24110937503993	0.16143862682111	1.23921449921167
H	-3.59487218726938	-0.56530490520691	0.62750007160843
N	0.73801886676605	-2.65646259694739	1.86769850323298
H	1.75021798354398	-2.70192791991574	1.86479865947658
H	0.43122314273222	-2.95901505370187	2.78602572995862

2^P^ E = -841.238761248 au
ZPE = 0.148817040 au

C	-1.82269740704427	0.01120534205765	1.29896531835273
H	-1.49097095000502	0.41351390280259	0.33748483986105
H	-1.55599657787528	0.74873717469486	2.06274727177252
C	-1.11843945276568	-1.31417948392357	1.59431331314929
H	-1.39781753026337	-2.05537911592037	0.83855453084521
H	-1.47419074643774	-1.70753711367107	2.55183316030686
C	0.40489534519215	-1.17636023852617	1.63088094851831
H	0.76680665368997	-0.80623890071860	0.66710754081111
H	0.70231913166159	-0.44861076812467	2.38785248945312

C	-4.16581642949254	1.52217655968610	0.69345742154792
H	-5.23412876237934	1.52470866785364	0.47243922042857
H	-3.98815611009738	2.26683963674436	1.47125689335192
H	-3.61735792414063	1.81275509225531	-0.20380227054981
P	-3.67835193375972	-0.14803498892486	1.33864968628680
H	-3.81358783881387	-0.85972768055333	0.11544547592810
P	1.23926368965969	-2.82408615899089	1.91944807731676
H	2.54895429158542	-2.33005021268200	2.15886965588017
H	0.94193751128603	-2.94189326405895	3.30361998673937

2^{As} E = -4628.931961979 au
ZPE = 0.144545130 au

C	-1.81366107310020	0.00694654835083	1.34758500790436
H	-1.52124541441657	0.42577000978881	0.38270656041186
H	-1.55031909903866	0.73762361432667	2.11683137838194
C	-1.09911713042936	-1.31747428087285	1.60561404048514
H	-1.38573859073158	-2.04347021263471	0.83755145905787
H	-1.43639154440762	-1.73525406817036	2.55983432114685
C	0.42040495124413	-1.16579746254618	1.62317305358185
H	0.77604832894804	-0.76943203574304	0.66927824878589
H	0.73486217833458	-0.47652968467341	2.40650744714912
C	-4.23836592373089	1.61007204955160	0.64675514418628
H	-5.29678639734286	1.63932437901341	0.39027612352979
H	-4.04546662388013	2.36741143630248	1.40631419550048
H	-3.64366353912101	1.82633077428381	-0.23941716585423
H	-3.90446098489837	-0.92476054164548	0.07854275132822
H	2.71255703654532	-2.34087287238453	2.16933562786152
H	1.00502394185955	-3.05125066126040	3.35880245332262
As	-3.80085198455479	-0.17262766711009	1.40847883397310
As	1.33383681872043	-2.92817087457656	1.87095407924734

2^{Sb} E = -638.989821684 au
ZPE = 0.139470670 au

C	-1.80046769341060	-0.01319944940341	1.39972874696009
H	-1.53253934833316	0.43002366145236	0.43908752522474
H	-1.53739798381756	0.70443615348576	2.18056900838661
C	-1.07209772143276	-1.33793058544052	1.61649185444064
H	-1.37610453768349	-2.05205153065791	0.84352476979066
H	-1.38222017828883	-1.77559874115640	2.57141114056315
C	0.44681024431105	-1.17757087284210	1.59820723872241
H	0.78238157439558	-0.77269285931780	0.64100836760670
H	0.78235178421430	-0.49976851345202	2.38279994944552
C	-4.36578236048792	1.75991980029401	0.60174991268724
H	-5.40857783862269	1.82876776971920	0.29531540559192
H	-4.17179553135768	2.51274171437202	1.36496227657306
H	-3.71994912122599	1.93407087041317	-0.25667369149968
H	-4.04273185585908	-1.00946049375002	-0.07322711765115
H	3.00850098220630	-2.35809104685225	2.18242573844673
H	1.13119049055153	-3.17943181102748	3.53879268985922

Sb	-3.98480040620585	-0.21295642004639	1.45234696355983
Sb	1.48989443104686	-3.09336921579021	1.86060277129229

2^Bi^ E = -587.489558644 au
ZPE = 0.136858380 au

C	-1.77387681324183	0.02448721034824	1.47151074769608
H	-1.54366521719026	0.50005213645889	0.51757605250037
H	-1.50670688996596	0.71845382309323	2.27121423743315
C	-1.04346793763383	-1.30564674216282	1.62625646755149
H	-1.36094223544987	-1.98686077401385	0.82915134430077
H	-1.33733267708157	-1.78213060990445	2.56786819362096
C	0.47301136675365	-1.14276102101719	1.59431074673604
H	0.80582316213653	-0.69936854237404	0.65412649782698
H	0.83106037535010	-0.52043058342019	2.41299831749147
C	-4.42965305448220	1.79753079979822	0.52917497581181
H	-5.47328149791751	1.85741169841308	0.22618869773255
H	-4.20830420553568	2.59475482433099	1.23719910699667
H	-3.78251630196446	1.88049207534170	-0.34074686679071
H	-4.08825742475283	-1.11200933767234	0.00201166002144
H	3.11047890677325	-2.41274824053566	2.23741914273765
H	1.08248478310272	-3.35354323499609	3.48567615499804
Bi	-4.05634122697720	-0.20202328744806	1.56246250446612
Bi	1.54815181807697	-3.16782176423966	1.75472557886911

2^O^ E = -308.477116836 au
ZPE = 0.141070690 au

C	-1.87374511149424	0.30701324002269	1.17554554788762
H	-1.51500859196557	0.67770950222875	0.20263006349952
H	-1.61424267176641	1.06876847982873	1.92716998084369
C	-1.22435008357146	-1.02144859177004	1.51347825505200
H	-1.50153664895255	-1.76184233670136	0.75964468193987
H	-1.60593292288144	-1.37749771061255	2.47314251937035
C	0.28750284612577	-0.92077015987891	1.58275775187646
H	0.68734915367943	-0.57180467030318	0.62104448815674
H	0.58242001256381	-0.19149784627883	2.34923615436852
C	-3.96906104883681	1.31355423630847	0.81086225701511
H	-5.03212300502704	1.07676596875516	0.80002918976312
H	-3.78648451742279	2.10311668093580	1.55233264885316
H	-3.67677549173841	1.69299159617616	-0.17769348197707
O	-3.27584977469781	0.12970186994905	1.13477489842054
O	0.79815883157265	-2.21349294715211	1.89843363464742
H	1.75821019441288	-2.16514612150783	1.94922316028295

2^S^ E = -953.711394510 au
ZPE = 0.132167780 au

C	-1.83887436365121	0.14786772305091	1.19910945778330
H	-1.49768470499179	0.51621575566223	0.22808330923787

H	-1.61614098402623	0.91158020440412	1.94876600194780
C	-1.13890087149345	-1.16167872909472	1.54886689191103
H	-1.39460528801240	-1.92359231479104	0.80779375999018
H	-1.50042687363858	-1.52370120786385	2.51487946772672
C	0.37539701062849	-0.98645329923892	1.60059864277851
H	0.75310912314634	-0.65395319988589	0.63312562229702
H	0.64759565298553	-0.24706702451371	2.35446856430482
C	-4.16505488863548	1.58584880029202	0.74462290055114
H	-5.25209641157950	1.57548756070140	0.67560761752857
H	-3.86892881567941	2.28070959948597	1.53150366450985
H	-3.75494292137792	1.91563739037922	-0.21082642070331
S	-3.64146617865071	-0.10470682632451	1.13418112523128
S	1.15222611152876	-2.59162157918523	2.02850986757767
H	2.42659241344757	-2.16790946307799	2.00330326732752

2[Se] E = -4959.095729878 au
ZPE = 0.129337840 au

C	-1.82561205944981	0.11929636896572	1.21466280334276
H	-1.51331264651295	0.49134597641412	0.23810398323185
H	-1.61785422205336	0.88446989140846	1.96364338212794
C	-1.11638464150058	-1.18608460738093	1.55513984311496
H	-1.36813939119185	-1.94558913184921	0.80986914140305
H	-1.47192991936295	-1.55724277198766	2.52017197484595
C	0.39441607846064	-0.99426495511226	1.60351296098658
H	0.77810823461741	-0.65699447880021	0.64208058994121
H	0.67410572454054	-0.27641523417014	2.37269000022448
C	-4.26313918554372	1.69156646708877	0.70967599927207
H	-5.34857070012609	1.72456711974144	0.64014820461439
H	-3.92943467065216	2.36957812054360	1.49271908254676
H	-3.82978705403528	1.97301600661953	-0.24807035485554
H	2.63674948335284	-2.18559443366132	2.03110594973091
Se	-3.77957963706928	-0.15563988709873	1.15927914298719
Se	1.26616260652663	-2.72335106072119	2.03786105648544

2[Te] E = -693.362783747 au
ZPE = 0.126562400 au

C	-1.80997703925528	0.06834053012767	1.20134524173523
H	-1.50321287563493	0.43639359430168	0.22280661479391
H	-1.62099327692503	0.84141288307656	1.94567406661846
C	-1.08936672964433	-1.22760113982382	1.55590680075692
H	-1.33142617015885	-1.99758548919124	0.81735474830413
H	-1.44520054324875	-1.59446226048564	2.52297787668356
C	0.41969308938837	-1.01890157745311	1.60719490623403
H	0.81009673392516	-0.69480161861857	0.64462446751577
H	0.69505932810860	-0.29409152723452	2.37060091571839
C	-4.39457405810712	1.84416131713680	0.68277083620451
H	-5.47666831199224	1.93932922571660	0.62138373681812
H	-4.01598509461648	2.47536931454086	1.48266639054160

H	-3.94752943723202	2.11607106781468	-0.26989044974715
H	2.93759133005068	-2.20318971300810	2.09658184364620
Te	-3.96480302657185	-0.22599730373121	1.12834080856545
Te	1.42309406191407	-2.89178392316864	2.11225494561088

2^Po E = -632.664480593 au
ZPE = 0.125055200 au

C	-1.78669332337754	0.05581073793133	1.17934463896555
H	-1.49265987186873	0.41465095034576	0.19467609223713
H	-1.61556950567269	0.83815005664745	1.91690371741928
C	-1.06851311088536	-1.23537234341240	1.54906498024641
H	-1.30545241057170	-2.01175215027248	0.81489549149098
H	-1.42979678800244	-1.59374032139931	2.51763397102891
C	0.43826765326050	-1.02401042888133	1.60766243504324
H	0.84568748920317	-0.71790334645468	0.64713622329035
H	0.71882348233291	-0.30484564030428	2.37342687127640
C	-4.47005304410879	1.91842428875173	0.67717297562451
H	-5.55189499453230	2.02439147354948	0.64418597219516
H	-4.05755088944671	2.52140783177848	1.48077354656061
H	-4.03537723584041	2.18546395831038	-0.28136265961382
H	3.06482991531843	-2.24763854630105	2.15650392774666
Po	-4.04411256973972	-0.26347812656176	1.10678212058777
Po	1.47586318393139	-2.98689499372730	2.15779343590086

3^B E = -222.832523076 au
ZPE = 0.170059260 au

C	-4.90430136555420	0.18373951512135	-0.13572084814101
H	-4.36348207158281	-0.66457967633714	-0.56618973230677
H	-4.75014350516522	1.01772454734320	-0.82845910279182
C	-4.28877309585797	0.53601221506397	1.22374973001852
H	-4.84300197266046	1.35621041032900	1.69755476934043
H	-4.43435327736311	-0.31903409512776	1.91091559278747
C	-2.08405299582202	1.73146436272761	2.31608326607071
H	-2.69075192717775	1.82007908799422	3.22506079026261
H	-2.19509314874403	2.71816408168901	1.82847310951933
C	-0.60803066571758	1.50308694549688	2.65791019376513
H	0.00323277895430	1.46876241817565	1.75354942077200
H	-0.20891944529478	2.28970075879989	3.30233250363316
H	-0.46830796784458	0.55276450466111	3.17866338439380
C	-6.39465113342212	-0.13994787141669	-0.06301674958097
H	-6.96143135901981	0.70388786521360	0.33947147953300
H	-6.80383273047921	-0.37648221682600	-1.04781902035254
H	-6.57713113266601	-0.99800563577365	0.58979267762376
B	-2.74631175489887	0.77338684431136	1.27639283759367
H	-2.04648437968374	0.20680663855438	0.48022723785949

3^Al E = -440.048428189 au
ZPE = 0.162980660 au

C	-5.12753434284896	-0.11090968948808	-0.18838141697261
H	-4.69499200812048	-1.11561433394084	-0.24372060297345
H	-4.79940892685096	0.39615629232411	-1.10209129836567
C	-4.57215693164667	0.62435064762695	1.04039196786655
H	-5.02097797255638	1.62623426165622	1.10568373736619
H	-4.90865625986377	0.12064780491269	1.95783692417258
C	-1.78497671959912	1.72138490377795	2.68426906922610
H	-2.21093037100421	1.28760744621809	3.59836885381985
H	-2.13896938353235	2.76170070989356	2.69234454723841
C	-0.25004205309187	1.69166406056006	2.75050148670079
H	0.19958452056079	2.15288405171714	1.86779170215131
H	0.13725245382192	2.22034288244804	3.62638629325615
H	0.13061048830928	0.66811551704659	2.79727247249807
Al	-2.61834293256725	0.83264935623062	1.13911767391041
H	-1.70339043345339	0.28960958274554	-0.04462469952471
C	-6.65192814612880	-0.22263518404024	-0.20008639596461
H	-7.11542908313782	0.76757242788031	-0.18931733527022
H	-7.01169903613512	-0.75121828894073	-1.08660660726630
H	-7.01070760215479	-0.76205260862797	0.68051860813113

3^{Ga} E = -2121.771854355 au
ZPE = 0.163059180 au

C	-5.13313447304295	-0.10753683042229	-0.19108272294384
H	-4.69478383209534	-1.10919699521928	-0.24922985734675
H	-4.79727241097429	0.41099763884256	-1.09515159266953
C	-4.60028178996047	0.61732518341041	1.04824370813042
H	-5.03415184924409	1.62279726419414	1.11706563376265
H	-4.93076248397665	0.10502077038233	1.96051616636636
C	-1.76839426948279	1.73139876111696	2.71043957120018
H	-2.17991942479695	1.28347891619917	3.62180627534634
H	-2.13854324456186	2.76339195957890	2.71215125292669
C	-0.23722916236216	1.70912669186151	2.74024911642210
H	0.18889073694810	2.17772780828528	1.85011509334798
H	0.16360191587226	2.23734532149908	3.61067876236092
H	0.14914663019366	0.68757402184066	2.77510266489115
C	-6.65717070623400	-0.22480485216709	-0.21782654398542
H	-7.12542130856189	0.76305803338234	-0.20276886293223
H	-7.00546148341623	-0.74654717720312	-1.11279911935977
H	-7.02257946140650	-0.77440420304639	0.65367794094351
Ga	-2.62018219909843	0.81404854260751	1.15859916649842
H	-1.70904591379937	0.23768898485729	-0.00413167295922

3^{In} E = -387.671245829 au
ZPE = 0.161675730 au

C	-5.25064125610390	-0.14780362354908	-0.24668660642061
H	-4.81644908583351	-1.15097181608618	-0.30918125818624
H	-4.89542797951246	0.38054268755444	-1.13766356512629
C	-4.74701420728854	0.55897206227198	1.01318998624421
H	-5.16329125858099	1.56953057445628	1.08185693527719

H	-5.09102958620579	0.03572307626188	1.91198723153426
C	-1.60003060865990	1.79619078101193	2.83499399721507
H	-1.96280472063253	1.33422240850999	3.75788250142820
H	-2.00036163281842	2.81435848030804	2.83709964263801
C	-0.07096748563867	1.80906971478616	2.79404755400180
H	0.30623182721282	2.28597934437347	1.88635252955251
H	0.35505939783938	2.35057960312083	3.64550915452581
H	0.34307348746911	0.79808396669398	2.81491338289867
C	-6.77535054746758	-0.25873642533499	-0.30818685629651
H	-7.24021667398122	0.73060647305135	-0.28996072483284
H	-7.10522106135196	-0.76712126289737	-1.21798773850340
H	-7.16126220120301	-0.81903567067235	0.54747301429326
In	-2.56768996608200	0.74225193728521	1.17081090076937
H	-1.56529684116075	0.02439153885440	-0.07195493101249

3^Tl E = -369.917661799 au
ZPE = 0.161557210 au

C	-5.27153624714725	0.05775200172711	-0.26765838262391
H	-4.74761455420123	-0.76260515873840	-0.76803183041225
H	-5.02131536767635	0.96187907776846	-0.83307449624990
C	-4.78046900196573	0.18680483178858	1.17255099363076
H	-5.26741630216421	1.01760010944217	1.68576814099804
H	-4.99924024921412	-0.72156766466713	1.74071640451517
C	-1.56988650332312	2.03594040013547	2.63604100748097
H	-2.09415992889085	2.01870954906993	3.59302121147221
H	-1.80115326868348	2.99549171661204	2.16729254097406
C	-0.06695346919155	1.84604466541362	2.80923061810439
H	0.45574936734126	1.85667244031981	1.85036521713407
H	0.36983527230972	2.63804680050053	3.42811019135803
H	0.16899692574884	0.89452083908809	3.29124852967393
C	-6.78134109706198	-0.17886203328176	-0.36049302325902
H	-7.33536498189640	0.63888846589933	0.10726045310018
H	-7.11082635243515	-0.25636139693233	-1.39984984344732
H	-7.06280937586628	-1.10312246659375	0.15051296282071
Tl	-2.55823186495255	0.48944841152811	1.33478348209364
H	-1.50619016072946	-0.55076389907993	0.34242823263620

3^Si E = -487.730627657 au
ZPE = 0.175798260 au

C	-5.04674992042599	-0.09241637418170	-0.13008496840430
H	-4.64324323957865	-1.10923521147196	-0.17228276884634
H	-4.71442425427741	0.40115807162338	-1.04915274695572
C	-4.45995923503258	0.63967970086182	1.08416603610974
H	-4.85919358059118	1.65979487806522	1.12965266559620
H	-4.78911202456544	0.14792246058313	2.00724298355480
C	-1.92673912387642	1.63912258350456	2.61402196626934
H	-2.30442894388366	1.13125827913229	3.50797114694913
H	-2.36581397739249	2.64235888524687	2.63093633379633
C	-0.39595614893310	1.73275180544471	2.66960299898473

H	0.00188260531831	2.26270270500179	1.80103354258548
H	-0.05684297412544	2.26371514779654	3.56219922194674
H	0.06257167462013	0.74110935777775	2.68326935828031
C	-6.57302775400235	-0.15530350660756	-0.11499482455240
H	-7.00596882197469	0.84843823868248	-0.10973205174406
H	-6.96060690517217	-0.68195022625726	-0.99011618217963
H	-6.93465148093846	-0.67521069067343	0.77587126898090
Si	-2.57996250322223	0.73321565607610	1.09808392291265
H	-2.10776873270108	1.42697136679569	-0.13199315113388
H	-2.01560596924508	-0.64447617740042	1.06787180784996

3^{Ge} E = -2274.547690308 au
ZPE = 0.174095140 au

C	-5.09809231758837	-0.10737543416525	-0.15016403879875
H	-4.69270943915109	-1.12354760842748	-0.18995000286052
H	-4.76939742235213	0.38439937802721	-1.07149490428220
C	-4.51650252135014	0.62836990577633	1.05898586859903
H	-4.90323425278125	1.65113718717839	1.10213193742696
H	-4.82834563268066	0.13834124115467	1.98646807170345
C	-1.86641752352331	1.67208351392252	2.65912910365800
H	-2.25723443207792	1.15734739572385	3.54083053397895
H	-2.30558243293823	2.67311334139696	2.66223975272646
C	-0.33954103975048	1.75386550951983	2.71765120455349
H	0.06588146949446	2.28115849617544	1.85094563782339
H	-0.00031728563570	2.28363493010014	3.61178110238789
H	0.11291077984445	0.75949385754762	2.73472147472506
C	-6.62491837148363	-0.17379179129399	-0.13158536355340
H	-7.06026401647456	0.82888324188920	-0.12980183518492
H	-7.01255490289746	-0.70508265053157	-1.00388305286536
H	-6.98348520646409	-0.69101535224423	0.76204034332636
Ge	-2.54513794247879	0.72643180022002	1.06392257907956
H	-2.05919111899599	1.45793010050551	-0.21156847526518
H	-1.95783461071507	-0.70619080247518	1.02994200282173

3^{Sn} E = -412.478703086 au
ZPE = 0.170923190 au

C	-5.22062413102947	-0.13796646605484	-0.20770479701750
H	-4.81874718321565	-1.15489791607472	-0.26207090622762
H	-4.89347965237405	0.36406498938511	-1.12404143322020
C	-4.64064898512673	0.58183141406344	1.01072961352847
H	-5.01462011809978	1.60764164226275	1.06613152473053
H	-4.94685507072823	0.08317600800376	1.93427580763232
C	-1.72158460424535	1.73612700288275	2.76336724700433
H	-2.10817257826262	1.21914501593094	3.64426157264029
H	-2.17380936182476	2.73014381028536	2.75571747853042
C	-0.19595078898081	1.82997229665653	2.81121917044383
H	0.20241877623093	2.35776855515462	1.94156566862041
H	0.14433476153966	2.36687201293994	3.70178915068026
H	0.26782602097972	0.84086831679914	2.83116896464016

C	-6.74836948850987	-0.20269894133480	-0.18948712315921
H	-7.18199139173401	0.80054041951507	-0.17378944769289
H	-7.13667317198655	-0.72161146186832	-1.06916419400987
H	-7.10679059124628	-0.73173647095504	0.69717256860938
Sn	-2.47168607149228	0.67935649061198	1.01914570718006
H	-1.92804663265292	1.47892600383789	-0.40738453136413
H	-1.82746116724091	-0.91906737204156	1.00100592845095

3^{Pb} E = -390.880284971 au
ZPE = 0.169462610 au

C	-5.26481119916092	-0.15015423210912	-0.22736701860462
H	-4.84895454942234	-1.16144492799944	-0.27569014096987
H	-4.92632463150159	0.36042357079951	-1.13448926472654
C	-4.72623891337152	0.57143677998807	1.00257817229898
H	-5.08879720870406	1.59954958688488	1.05401465776493
H	-5.01893593781756	0.06540657973734	1.92423983416115
C	-1.64047656301991	1.78176537527140	2.82911604174146
H	-2.02464326262412	1.25904308170423	3.70535036109751
H	-2.08535427574965	2.77682799375341	2.80918486793732
C	-0.11783041828342	1.84112966023303	2.82826833770966
H	0.26561264267293	2.35937306419099	1.94661316843221
H	0.25771043217041	2.37366045658765	3.70831162553809
H	0.32502316181486	0.84264229888065	2.83939675718931
C	-6.79294213568431	-0.23494117495263	-0.24010619793055
H	-7.24014995160756	0.76225525415987	-0.23004636486276
H	-7.15435358219301	-0.75542576197059	-1.13018377772656
H	-7.16228022969394	-0.77305684732359	0.63639642177880
Pb	-2.46930989460920	0.68722545532892	1.03061757488903
H	-1.91112616599759	1.50710107315948	-0.45214163338108
H	-1.80270467721746	-0.96719163632407	1.00433912766354

3^{N} E = -252.728026077 au
ZPE = 0.177106470 au

C	-4.73158943390638	0.03524302297452	-0.00135984228116
H	-4.29426285101218	-0.96655071014528	-0.01897060911237
H	-4.35669486607462	0.55331301149462	-0.89184224727633
C	-4.23473854177263	0.76062613393711	1.24211382395180
H	-4.67750041530485	1.77315972958826	1.27541657715492
H	-4.59632218789588	0.24002756057989	2.13587398927970
C	-2.25292260439020	1.51433793307964	2.44868893224284
H	-2.60572171558160	1.00794448077135	3.35291235780414
H	-2.63909489360455	2.54779526566001	2.50316423390867
C	-0.73221039348317	1.53708979345441	2.43552982848339
H	-0.35459799447872	2.05264669405052	1.54796733101628
H	-0.34397454473882	2.06031013221905	3.31145567391201
H	-0.33250028232394	0.52164598740714	2.43062129296973
C	-6.25416829930107	-0.04945940880083	-0.06470974941692
H	-6.70791887077065	0.94496240617581	-0.06964646211588
H	-6.58546896254073	-0.56931702313619	-0.96540560766358

H	-6.65439052439605	-0.59057713610058	0.79644969117651
N	-2.78108969084791	0.78999610036268	1.30129533914833
H	-2.41397079757603	1.19570862642787	0.44623546681787

3^{P} E = -538.972574878 au
ZPE = 0.169186510 au

C	-4.94285884423276	-0.06736387007131	-0.12085730331966
H	-4.63573872785668	-1.11496801962782	-0.19421966180703
H	-4.55262326970815	0.42730764378507	-1.01625760268021
C	-4.30819368459699	0.55940190155909	1.12121519916025
H	-4.59582321931656	1.61155265285922	1.20538902682362
H	-4.68052176006817	0.05459440981484	2.01883177539350
C	-2.02372647632159	1.50108785089280	2.56790269532704
H	-2.36367627909998	0.99298736683771	3.47508254216368
H	-2.58064702409567	2.43913787247096	2.50068104270603
C	-0.52159835114115	1.77132075372070	2.65353963776138
H	-0.16380127530469	2.29958517000757	1.76683826428971
H	-0.28201571565971	2.38618326820502	3.52392878319871
H	0.04448447096438	0.84097061373214	2.73403311667995
C	-6.46780574337747	0.02148623355640	-0.11635741848698
H	-6.80236609228529	1.06141989068922	-0.08188334016619
H	-6.89431046892667	-0.43607544287708	-1.01155210058899
H	-6.88852043772091	-0.49041645903124	0.75283765482140
P	-2.45483883174481	0.38633362883940	1.13924964028260
H	-2.18398284950710	1.32452872463729	0.10460009844118

3^{As} E = -2432.818701847 au
ZPE = 0.167156230 au

C	-4.74813729426170	-0.07546892460766	-0.40014812198326
H	-4.45148085240437	-1.12383901376866	-0.50502468714740
H	-4.22220769548478	0.46935506167919	-1.19075873226291
C	-4.29991430451260	0.44716750360008	0.96172362507597
H	-4.55197320989050	1.50351135688589	1.07634846203528
H	-4.80521719195131	-0.09919677631643	1.76304045520621
C	-2.17478574734551	1.36715736919850	2.88066441136347
H	-2.65331709703956	0.82449071896793	3.69907987171852
H	-2.74359550965206	2.28376929233031	2.71795718053457
C	-0.71859248184070	1.67339145930666	3.21642343474877
H	-0.23446469225405	2.23095937758158	2.41133772453514
H	-0.64074639546507	2.27464167139526	4.12603375503213
H	-0.14356625180214	0.75828398681550	3.37462660940938
C	-6.25614509163794	0.05037407676500	-0.61573391225268
H	-6.57675645773400	1.09341412012720	-0.55429234889500
H	-6.55052537319680	-0.33173911931678	-1.59572926911769
H	-6.80901428998706	-0.51102418910252	0.14166792369148
As	-2.34455143796304	0.20344478020220	1.26789082177005
H	-1.91556344557679	1.29015777825674	0.27644817653795

3^{Sb} E = -437.847271473 au

ZPE = 0.164846300 au

C	-4.81187526078527	-0.19769786693348	-0.45115559190279
H	-4.51947604797874	-1.23923713607922	-0.62030100437574
H	-4.31246668536639	0.38697077691762	-1.23037286129761
C	-4.32481816406304	0.25817093982961	0.92131594366905
H	-4.56795043640391	1.30791187509694	1.09642999437479
H	-4.79992291839854	-0.32656976999435	1.71346256200041
C	-2.04362161792082	1.30566059019364	2.97029298036133
H	-2.45866365273953	0.72357520365160	3.79564320067112
H	-2.70329468274538	2.15777963445054	2.80294996252654
C	-0.61909066930307	1.75591508913618	3.27968747091304
H	-0.20271313643401	2.35142533408400	2.46405486330315
H	-0.58360514693547	2.37005637329608	4.18433041937900
H	0.04812057706409	0.90502239830574	3.43709945455931
C	-6.32676564404593	-0.06772299061890	-0.61734742999225
H	-6.64735211581451	0.96974863390037	-0.49382457187973
H	-6.64864939570626	-0.40146161907622	-1.60665794544192
H	-6.85542722226463	-0.66843920321838	0.12703784384843
Sb	-2.16299436558837	0.02402076816250	1.19417914515090
H	-1.78446362457020	1.31524537889573	0.11860652413294

3^{Bi} E = -412.096173031 au
ZPE = 0.163610180 au

C	-4.82398555161211	-0.27653081941780	-0.46795417018523
H	-4.52417376301522	-1.30446214773467	-0.69858450151482
H	-4.34233740059495	0.35516736898127	-1.22097588025966
C	-4.32575678889170	0.11039607513346	0.91880519191120
H	-4.56308344259073	1.14842527768989	1.15543495741694
H	-4.77087858003058	-0.52192323565012	1.69070575575735
C	-1.96112691527715	1.22846592199255	3.01306154621462
H	-2.34164187672550	0.62181213808394	3.83660055449872
H	-2.66341661075397	2.04350361510843	2.84079237569521
C	-0.55498790178257	1.74034261609527	3.29644347171721
H	-0.17817147661660	2.35139015970132	2.47320329295443
H	-0.52930268824598	2.35873696142501	4.19975107740852
H	0.15282154457979	0.92112403906789	3.44648511021157
C	-6.34351025457790	-0.15675214723411	-0.60930768860683
H	-6.67388137991775	0.86851323887321	-0.42513307945369
H	-6.67375766642731	-0.43978859752919	-1.61180111675858
H	-6.85571411667336	-0.80342481998892	0.10752974017680
Bi	-2.05687803187717	-0.11502182120979	1.15528295554211
H	-1.73129507896922	1.27708975661237	0.05080533727415

3° E = -272.588213990 au
ZPE = 0.164359190 au

C	-4.42294996437403	-0.10988329439514	-0.11828051675177
H	-3.92265853629508	-1.08129755596924	-0.15682631718559

H	-3.94768025477205	0.51793185032512	-0.87682490323974
C	-4.17472882563831	0.50798228369065	1.24462021551341
H	-4.65443829049349	1.49712047213573	1.30895946578473
H	-4.61879061305961	-0.11785669608844	2.03440140105434
C	-2.44690080916619	1.20443425324087	2.70030235190697
H	-2.86393312717424	0.59400005641380	3.51511822973386
H	-2.89499757754090	2.20580854771360	2.78294488628037
C	-0.93916951863001	1.28456527805021	2.81214441599310
H	-0.52717012090423	1.89688683952769	2.00846423954725
H	-0.65249928110695	1.72872923424586	3.76754528708966
H	-0.49572750248711	0.28967407780447	2.74666854780741
C	-5.91146309098120	-0.26810967239055	-0.41807106980412
H	-6.42466010567902	0.69683521124722	-0.40611318571911
H	-6.06876378394902	-0.71355306872710	-1.40162061907936
H	-6.39954635800823	-0.91187467698006	0.31815850119756
O	-2.78176694974033	0.63139170015531	1.45066861987103

3^S E = -595.207628231 au
ZPE = 0.160936770 au

C	-4.63033505160962	-0.18981810564164	-0.31287532807411
H	-4.17524895275895	-1.17947556458160	-0.40985494238974
H	-4.23461890363779	0.41600637578100	-1.13289955773390
C	-4.20759834744983	0.42692160955576	1.01619601093004
H	-4.62739181160308	1.43129417375358	1.11883168685941
H	-4.57557573376054	-0.17861496346829	1.84893848168008
C	-2.22077667994069	1.26367473572517	2.78242866270632
H	-2.72622110962496	0.61735023380150	3.50371765796260
H	-2.72075123869955	2.23498175323106	2.79121373893825
C	-0.74779265484828	1.41537079979056	3.13864339576820
H	-0.23385267070289	2.05873753892296	2.42217948739918
H	-0.64185121432484	1.86123735283327	4.12981498754201
H	-0.24350013050464	0.44759637299674	3.14670318018783
C	-6.14843420322845	-0.30343321834971	-0.43868644946988
H	-6.62589085768075	0.67750646942688	-0.37403493437374
H	-6.43035327673397	-0.74757461192372	-1.39520941696654
H	-6.56550170152736	-0.92907456019164	0.35431394488379
S	-2.39215017136381	0.53009844833811	1.12283895415019

3^Se E = -2597.899881926 au
ZPE = 0.159732640 au

C	-4.69956125217367	-0.21212582355276	-0.36798006483547
H	-4.25011978508374	-1.20357170560949	-0.47380036354930
H	-4.32706626511852	0.38873448124621	-1.20263601526916
C	-4.24567599922630	0.40964263378579	0.94572714156270
H	-4.64593341243197	1.41832544698243	1.05839591406135
H	-4.57433611347009	-0.19150478694943	1.79490393396105
C	-2.14029958687030	1.30074845780507	2.83112726592246
H	-2.67481729273548	0.63837310920961	3.51225697497182
H	-2.64505129756797	2.26679401270234	2.81748755777040

C	-0.68166610137265	1.44636439819558	3.23612261678585
H	-0.14049978698954	2.09597515384980	2.54552438373169
H	-0.60676159290029	1.88412299132272	4.23456103679682
H	-0.17706099677317	0.47879197977272	3.25519488945950
C	-6.22137971745169	-0.32597041985779	-0.45628711431054
H	-6.69627243691308	0.65572592668346	-0.38549935410940
H	-6.52634072141844	-0.77560560418967	-1.40333389472904
H	-6.61853954593601	-0.94685855612676	0.35048193332725
Se	-2.27646282556705	0.52482312473017	1.02001273845202

3^Te^ E = -465.033479064 au
ZPE = 0.158613730 au

C	-4.79497175686219	-0.24536386880002	-0.44267992266404
H	-4.35694616064584	-1.24057912547634	-0.56247601296640
H	-4.44966057767429	0.34933463171798	-1.29366123616120
C	-4.29538065711556	0.37861796021423	0.85362672508191
H	-4.67782734615814	1.39172384722257	0.97730947470151
H	-4.59161072077922	-0.21645261637653	1.71758637944053
C	-2.02859709414846	1.34844679245493	2.89435828007527
H	-2.59520907610821	0.67914220448188	3.54022904287953
H	-2.53473515818866	2.31180617352848	2.85678317681368
C	-0.58880959651448	1.49132950988543	3.36488879536719
H	-0.01435292653278	2.14597558868512	2.70627090986378
H	-0.55599236530790	1.92299607954559	4.36906968175900
H	-0.08238625914985	0.52502776228622	3.40225881595062
C	-6.32006836012399	-0.35234303941009	-0.48270724625876
H	-6.78668654380250	0.63226108244577	-0.39916943149372
H	-6.65742615521311	-0.80318461679010	-1.41846242508975
H	-6.69322870262222	-0.96878472191378	0.33870462811965
Te	-2.12395527305257	0.50283117629868	0.89032995458119

3^Po^ E = -434.683900483 au
ZPE = 0.157939420 au

C	-4.83955873895163	-0.25734635669275	-0.47178093304102
H	-4.39401404532914	-1.24973645679811	-0.58869155332523
H	-4.50893178779805	0.33406945883417	-1.33112272264770
C	-4.33813017583286	0.38035613299074	0.81440531601930
H	-4.71644578213685	1.39406983064026	0.93735932777230
H	-4.60474998596718	-0.21149769261992	1.68903915894210
C	-1.97492917910392	1.39076880387458	2.93540574727633
H	-2.56627686019570	0.71754982748324	3.55294138955982
H	-2.46529268328910	2.36082793076052	2.89099254348330
C	-0.53969591775565	1.49715689078661	3.42142904759723
H	0.05616229268411	2.15084009714957	2.78074552548981
H	-0.50655128370542	1.91337753462829	4.43315250257156
H	-0.05179602414080	0.52092866541300	3.45080021434629
C	-6.36482676315728	-0.38094758491108	-0.50113861822747
H	-6.84079253374044	0.59937285403851	-0.42120904854779
H	-6.70328205871002	-0.84214022997835	-1.43167526148030

H	-6.72538923534737	-0.99531315447493	0.32731332726761
Po	-2.06334396752269	0.53044825887565	0.82429362694385

CH_3BH_2 E = -65.815906787 au
ZPE = 0.055506120 au

C	-0.59346352662657	0.29071562992790	-1.41972648837236
H	-0.32755335198140	-0.76691661491994	-1.46522390906419
H	-0.33773381441444	0.78329450332802	-2.35936632427845
H	-1.69607180737253	0.31752625777361	-1.35641959570987
B	-0.12423058164531	1.03013984721956	-0.13824365626648
H	0.02159204747025	0.44060625152328	0.89081040812075
H	0.04077580456999	2.21354698514756	-0.13582862442941

CH_3AlH_2 E = -283.041023753 au
ZPE = 0.047677800 au

C	-0.64677143493066	0.29921916911832	-1.42239715816779
H	-0.17450148032405	-0.68167923095794	-1.53352183450978
H	-0.48393840004505	0.86379176673761	-2.34258066068219
H	-1.72343544345435	0.11028569608822	-1.33918194602254
Al	-0.00241498898158	1.22240092590588	0.17638209004199
H	0.05177132329238	0.45416304767513	1.56168488158060
H	0.45594704444331	2.73840385543277	0.12403154775971

CH_3GaH_2 E = -1964.764693469 au
ZPE = 0.047874810 au

C	-0.64436320959647	0.28203013779797	-1.43220127346308
H	-0.32720161966713	-0.76097051831778	-1.45285758306448
H	-0.31821854697775	0.77922497619426	-2.34629884971329
H	-1.73934235699038	0.29242044434431	-1.42584680701412
Ga	0.00405921490231	1.21767003696314	0.18755758372597
H	0.25659304237265	0.42118527867135	1.52386254908805
H	0.24513011595676	2.77502487434675	0.17020131044095

CH_3InH_2 E = -230.668436631 au
ZPE = 0.046074670 au

C	-0.64359491028641	0.30496264439500	-1.40519851070509
H	-0.16237096903460	-0.66934046747422	-1.49661769664916
H	-0.45775408644843	0.88819596085452	-2.30565132994128
H	-1.71887639090064	0.13847071931671	-1.31099805352100
In	0.07518518161573	1.33267047289303	0.37087374684137
H	0.18158919494027	0.47661126777096	1.87970381473049
H	0.53581772011407	3.00641487224399	0.30880164924467

CH_3TlH_2 E = -212.916177260 au
ZPE = 0.045708280 au

C	-0.65931599697372	0.29158152035981	-1.39386341984505
H	-0.28640629767181	-0.73006785316105	-1.40861883459652

H	-0.28570634872206	0.82257809496110	-2.27011341701404
H	-1.74708971643118	0.30005904944586	-1.40893830023952
Tl	0.08479543870042	1.34791148063199	0.41945215732090
H	-0.85412381054101	2.64951432677745	1.13424352950088
H	1.62451027163937	0.89068891098484	1.13205124487334

CH_3CH_3 E = -79.698908710 au
ZPE = 0.074356700 au

C	-0.66960569408304	0.27781712973649	-1.43976949301436
H	-0.32394864249805	-0.75779177434643	-1.47006255399541
H	-0.32155031201149	0.76800117082123	-2.35167291665441
H	-1.76131165647110	0.26106146920187	-1.47124472687837
C	-0.16087537011529	0.99774689528094	-0.19296046905586
H	-0.50893148476619	0.50756369936174	0.71894312604490
H	0.93083054587662	1.01450208925591	-0.16148476638837
H	-0.50653164593146	2.03335606068825	-0.16266832005811

CH_3SiH_3 E = -330.719123454 au
ZPE = 0.060826650 au

C	-0.66124162996107	0.28959628149523	-1.41954321948295
H	-0.31107831788989	-0.74425311485672	-1.44257569641299
H	-0.31005074162623	0.78570412414433	-2.32658011655775
H	-1.75281495785858	0.27638666285826	-1.44342946132738
Si	-0.03583333157898	1.17446741761422	0.11325193825034
H	-0.51802550776460	0.49644922371806	1.34227863792445
H	1.44794219625719	1.18743506974239	0.14034176558809
H	-0.51414355957783	2.57927165528421	0.13832984201819

CH_3GeH_3 E = -2117.538759817 au
ZPE = 0.059138300 au

C	-0.66890294904389	0.27877351321535	-1.43841570930355
H	-0.31275551835265	-0.75114610190400	-1.45044343851174
H	-0.31285885007360	0.78273699931481	-2.33681230140865
H	-1.75875764008826	0.27129572908014	-1.45083236612554
Ge	-0.01361339890968	1.20579007684699	0.16722502010254
H	1.52730553542165	1.21503031343425	0.18914334966042
H	-0.51371532234815	2.66350641758082	0.18716307918945
H	-0.51861317660543	0.49692007243164	1.43916973639707

CH_3SnH_3 E = -255.472495713 au
ZPE = 0.055566340 au

C	-0.67089219442153	0.27586712311351	-1.44338369020046
H	-0.31053380235753	-0.75180368294279	-1.44929729983840
H	-0.31230407331188	0.78472538479444	-2.33701886052273
H	-1.76001850507688	0.27094846215820	-1.44926321943898
Sn	0.04797192333422	1.29401657369500	0.31934548766605
H	-0.51741968390831	0.49955444271831	1.72963997313191

H	1.76240972734896	1.29909882784716	0.33625333978338
H	-0.51111951160705	2.91476015861616	0.33476854941923

CH_3PbH_3 E = -233.875145805 au
ZPE = 0.053987900 au

C	-0.66001006886098	0.29107208266521	-1.41697649017913
H	-0.29208005657977	-0.73204952544891	-1.40486051677069
H	-0.29102988977135	0.81188512866630	-2.29733998887595
H	-1.74740017570600	0.29767963036808	-1.40639320190282
Pb	0.08612442111356	1.34821537605058	0.41369022352500
H	-0.49677299630869	0.52732465507494	1.86795897801786
H	1.85486726490385	1.35336994762004	0.42824407070564
H	-0.49226726879061	3.01965012500376	0.42826885548009

CH_3NH_2 E = -95.728601383 au
ZPE = 0.063693260 au

C	-0.65953278631415	0.26242234050062	-1.43112193811656
H	-0.33844801387170	-0.78634463335464	-1.50320641522639
H	-0.31178940099566	0.78126787967972	-2.32625763285235
H	-1.75082629558284	0.27995537134297	-1.45131051795292
N	-0.18792437647337	0.97802189954155	-0.24536923131315
H	-0.50048412934042	0.51645279720680	0.60067944769882
H	0.82463810257813	0.99456320508298	-0.21468014223746

CH_3PH_2 E = -381.964771067 au
ZPE = 0.054189140 au

C	-0.65899118668495	0.29424150014476	-1.41218661336002
H	-0.27803441659894	-0.72488057388612	-1.37368710660391
H	-0.30856812608155	0.77031871479751	-2.32906942287164
H	-1.74877247896624	0.26505726809662	-1.45111138922507
P	-0.14058244940862	1.34031555827732	0.03899863449502
H	-0.44531310895257	0.40788791181420	1.06555496095174
H	1.23888269669288	1.00325077075570	0.03354348661388

CH_3AsH_2 E = -2275.811415154 au
ZPE = 0.052018810 au

C	-0.68435596155684	0.28035265443571	-1.46114296461490
H	-0.28709606872560	-0.72870920149164	-1.38683736696175
H	-0.32352521233503	0.74889523539932	-2.37603973458529
H	-1.77249348070707	0.24339842124485	-1.49574528683998
H	-0.46092036109640	0.37831788573360	1.15482886370822
H	1.33037699535611	1.01515711197901	0.05533285086192
As	-0.14336498093516	1.41877904269916	0.08164619843178

CH_3SbH_2 E = -280.840083923 au

ZPE = 0.049297730 au

C	-0.72402243063980	0.26107804731569	-1.53662381746478
H	-0.31069232403737	-0.73826485436735	-1.42915368995835
H	-0.35772791416899	0.71544880085706	-2.45608503487095
H	-1.81109252416737	0.20953689180460	-1.57447968377061
H	-0.49794050329960	0.33335086049749	1.32630107184273
H	1.50389704083677	1.04587180553768	0.09685431362918
Sb	-0.14380041452364	1.52916958835482	0.14522939059278

CH_3BiH_2 E = -255.090408122 au
ZPE = 0.047923940 au

C	-0.74286464952172	0.24917785927710	-1.57368559146707
H	-0.32015113702716	-0.74249054840288	-1.44226109278488
H	-0.36769542761792	0.70135062252870	-2.48985683427609
H	-1.82933771336077	0.19593125600921	-1.60678824498128
H	-0.51062000265957	0.30963006827273	1.39425858048715
H	1.57442668015900	1.05509706994467	0.11178776292390
Bi	-0.14513681997187	1.58749481237046	0.17858797009827

CH_3OH E = -115.589719887 au
ZPE = 0.050972800 au

C	-0.78492534728666	0.22440621959889	-1.65763724041223
H	-0.40491083386639	-0.79603921222057	-1.62667117983160
H	-0.42347014972766	0.69938923341265	-2.57692054062010
H	-1.87982392995779	0.18394752555283	-1.68717878648387
O	-0.30384396063333	0.87750616712080	-0.49091398003025
H	-0.61830380852817	1.78710447653541	-0.49282859262193

CH_3SH E = -438.203424188 au
ZPE = 0.045805720 au

C	-0.78399573515936	0.19643431574237	-1.62962879749324
H	-0.40972368186705	-0.82546565354907	-1.63095180754012
H	-0.42257595711252	0.70338308020249	-2.52315620289926
H	-1.87229196649204	0.19975255944584	-1.64140801965209
S	-0.13257030109870	1.14028670011407	-0.21112119240623
H	-0.67798530827032	0.38360280804430	0.75534150999094

CH_3SeH E = -2440.895000307 au
ZPE = 0.044293690 au

C	-0.79825727674868	0.17619878928368	-1.68136066970296
H	-0.41846896811022	-0.84112421954252	-1.64992686734464
H	-0.44505567231624	0.66914688892291	-2.58455684666925
H	-1.88445676406703	0.18625166197957	-1.66256350411912
H	-0.67630517086893	0.38299128544008	0.87323546328332
Se	-0.07659908788890	1.22452941391628	-0.17575209544735

CH_3TeH E = -308.027714542 au
ZPE = 0.042664380 au

C	-0.82032849408176	0.14623121936181	-1.74540908997617
H	-0.43956877462723	-0.86788887375873	-1.67335836089604
H	-0.48382402929070	0.60290907407340	-2.67358244068732
H	-1.90465233442970	0.16368609781503	-1.69596823698607
H	-0.65543987327114	0.40135953821866	1.05160042956634
Te	0.00467057570053	1.35169676428983	-0.14420683102074

CH_3PoH E = -277.678380844 au
ZPE = 0.041817470 au

C	-0.83526273933431	0.12360112736858	-1.77785515566766
H	-0.44879879278017	-0.88556535014398	-1.68139086924985
H	-0.51039315239039	0.56783398129278	-2.71557379880309
H	-1.91691889684975	0.14769576299202	-1.69925875353386
H	-0.64352939916421	0.42956408096513	1.13154121706578
Po	0.05576004051883	1.41486421752547	-0.13838716981131

***c*-C_2P_2** E = -761.522657332271 au
ZPE = 0.07363564 au

C	-0.26998946405142	2.13204218348524	-0.20337531986226
H	-0.34648057703497	2.59379513884617	-1.18790030448146
H	0.54442873151114	2.59688644234657	0.34774387269273
P	-0.16877242509796	0.26064843864612	-0.40810358067496
H	0.09243830259428	-0.00045730573923	0.96661680370573
P	-1.90374858315192	1.99559745024030	0.69610016264225
H	-2.73622369201558	2.82797124523778	-0.10742968929105
C	-2.04015867311394	0.36177151059890	-0.20325886163386
H	-2.50198080041466	0.43817062154425	-1.18775861084834
H	-2.50491965321217	-0.45263748186136	0.34794431137942

PHD^{C-P}-*c*-C_2P_2 E = -1143.511253092921 au
ZPE = 0.12951180 au

C	-5.64593805193275	1.88084592134877	3.03753614427042
H	-5.16607907359346	0.91299868010931	3.19181758311463
H	-5.26874251665300	2.58515455693750	3.78027122398684
H	-6.71852770521320	1.74677694464492	3.18730128444772
C	-3.54285494346211	2.89656294690947	1.40118024490612
H	-2.96296457295466	1.97054745814541	1.42327559148951
H	-3.33976115008387	3.44756432278343	2.32281848113312
C	-1.16965611346638	3.79728772062751	0.08554764151612
H	-0.88394011503221	2.75422756467213	0.23840516674963
H	-0.83157656485307	4.37513551735679	0.94629037283432
P	-3.02067608134194	3.96834994528854	-0.02676121358074
H	-3.21890056363343	3.00671090316244	-1.05122580329064
P	-5.34395033048881	2.43328416517307	1.29152756508927

P	-0.34928851177991	4.38127128276876	-1.48854984544367
H	-5.86335201189031	3.74764550913586	1.42788155572173
H	0.96441535839454	4.52523227530001	-0.96787159811136
H	-0.64710364201521	5.76166358563587	-1.34929236483307

***c*-C_2O_2** E = -228.738120014356 au
ZPE = 0.06279454 au

C	-0.40864281263123	1.89386954780498	-0.00008228805363
H	0.00891800160093	2.35419092844626	-0.90325724616395
H	0.05172030932065	2.31151026240628	0.90303454624075
C	-1.80039185497500	0.50205940908317	0.00001447670735
H	-2.26074022765540	0.08440580272488	-0.90308996928475
H	-2.21795957286845	0.04175864310114	0.90318299712484
O	-0.37299283164779	0.46648672695480	-0.03460621696697
O	-1.83602487114371	1.92945941947849	0.03453455039637

PHD^{C-O}-*c*-C_2O_2 E = -344.373405030377 au
ZPE = 0.11771371 au

C	-4.92802977994134	2.57123109433312	2.66381436050731
H	-5.42437244972603	1.63117043786910	2.38982119209777
H	-4.31557501472145	2.39402933586737	3.55772777552927
H	-5.68664374295621	3.31631459873119	2.89625489740708
C	-3.12018706999186	2.19836194949505	1.20808734729499
H	-3.53899749363089	1.26542056511599	0.81302658363537
H	-2.48743660636134	1.97518133256561	2.08631513654643
C	-1.60788380049217	3.90002086657046	0.58315277730829
H	-1.10485007424909	3.69437438184627	1.53408871904931
H	-2.27288097530787	4.76297116545075	0.69243422804068
O	-4.15493347232313	3.07952591984399	1.59679646904407
O	-2.37651150276045	2.76667598629103	0.19155756262345
O	-0.61402911115117	4.12203587584636	-0.36135166234449
H	-1.02898589135410	4.38479324170408	-1.19136202389325

***c*-C_2ON** E = -208.873515533014 au
ZPE = 0.07549912 au

C	-1.78574043534895	0.44571939191509	0.03339008635104
C	-0.36011006446694	1.87175348774833	0.03387658146327
H	-2.14799192980074	-0.02951589250227	-0.88720606762170
H	-2.36375838549697	0.08285506798882	0.88900912867238
H	0.11533602880001	2.23445337369179	-0.88643415160092
H	0.00229874390605	2.44958243963584	0.88981617544322
O	-1.79521909027534	1.88085272096084	-0.04495729867600
N	-0.32551761292644	0.41147349438020	0.23010715552469
H	0.16323891359673	-0.07689247577802	-0.50928185723643

PHD^{C-N}-*c*-C_2ON E = -304.643676500968 au
ZPE = 0.14211442 au

C	-5.32969418448713	2.25218552404072	2.61883270415324
H	-5.01388998046069	1.19950647407810	2.70772176100226

H	-5.06401855958990	2.75668797254258	3.55126924129990
H	-6.41581750260915	2.27478405370742	2.52892579533782
C	-3.30036737790616	2.92248645453069	1.49888131054176
H	-2.89060578842430	1.88986789610333	1.48145103110586
H	-2.94059710563614	3.39944771458211	2.42439298253330
C	-1.44369679990945	3.63944930105771	0.25018267647227
H	-1.05341037011505	2.62185324283723	0.10850830592554
H	-1.02917320794310	4.02961496165371	1.19694321899034
O	-2.85293774204825	3.61737358583857	0.36196876200810
N	-4.73825984259108	2.96044382509939	1.49556788501682
N	-1.10722636528812	4.41938719230847	-0.90824161897575
H	-5.07514231137517	2.58938697935905	0.61450542109780
H	-0.18232118782211	4.20254351960492	-1.25168596719633
H	-1.17444087379413	5.41326356265596	-0.73193079931292

PHD$^{C\text{-}O}$-*c*-C_2ON E = -324.511279519693 au
ZPE = 0.12986053 au

C	-5.15130378919371	2.03727122137675	1.92797135275845
H	-4.68035385945421	1.09358838508432	2.23689989032915
H	-5.54544011200894	2.53455186505482	2.82580189435094
H	-5.97901123155395	1.81379206090996	1.25660470836787
C	-3.12838314041087	3.23581902693481	2.01886847917239
H	-2.58631347016222	2.33407639373920	2.33677000308023
H	-3.48875256927499	3.75356224243831	2.91885515847700
C	-1.37214329788144	3.53294758980456	0.32835564305662
H	-0.95034282150713	2.60258021413860	0.72697026687331
H	-0.55272593781549	4.23598604769953	0.13962862821116
O	-4.25058427183613	2.86324471576020	1.22664745800657
O	-2.07116864430700	3.27820244401441	-0.89609095423094
H	-1.42240234869587	3.10056410120954	-1.58730155867032
N	-2.22397951835418	4.08647816047197	1.32571510329547
H	-2.65049069754362	4.95629238136273	1.03444495692198

***c*-C_2PO** E = -495.115579066024 au
ZPE = 0.06818190 au

C	-0.95429973930004	1.68930624238753	3.52337247971388
C	0.59849079108715	3.22321465088673	3.54135699278661
H	-0.92923150480775	1.05669370035465	2.62947436227319
H	-1.62256986441036	1.24767480941481	4.26243331621967
H	1.24030072885019	3.20051914946131	2.65386776785083
H	1.04101625894009	3.87757247998293	4.29227928479101
P	-1.21419948116646	3.51090927954579	3.09464580234270
H	-1.60687608654583	3.89230020481169	4.40643200970641
O	0.36581247735305	1.90000690315439	4.05199567431546

PHD$^{C\text{-}O}$-*c*-C_2PO E = -610.734091247072 au
ZPE = 0.12124529 au

C	-3.83581387854536	0.22933901900057	0.87039199360335
H	-3.85291775402682	1.24868917261340	0.46391863383352

H	-4.38877078687574	0.23011245402912	1.81645910534866
C	-1.68432552509442	1.00229962909355	2.46831420520844
H	-2.40366657832940	0.93831538483906	3.29642241991678
H	-1.76775216488509	2.00957902290319	2.03283671753741
C	0.03217408895130	1.67156439699151	3.93208607339705
H	-0.61997231727679	1.61050029896746	4.81318897930922
H	1.04797642084590	1.40470436989143	4.22026241868722
H	0.02266212221978	2.70484427700592	3.56127819139256
O	-4.38514825826587	-0.70757214924479	-0.05001452976470
H	-5.30838422786411	-0.48346818460513	-0.20900102214558
O	-0.37163507740828	0.76293583569009	2.92898599935050
P	-2.04560427450380	-0.24586060718364	1.12537953880852
H	-2.29523145894126	-1.34291395999179	1.99111032551704

PHD$^{C\text{-}P}$-*c*-C_2PO E = -877.113604926961 au
ZPE = 0.12442755 au

C	-4.54759306646849	0.49082805895225	1.79376128921487
H	-3.76131197208407	1.25567025122925	1.72265996788158
H	-5.50910752043919	1.00199784453274	1.64372183517505
C	-4.68679628979938	0.74912762780595	4.13588500497161
H	-3.90100243122101	1.51644775109004	4.13455922773645
H	-5.65626365032199	1.26373673014344	4.06471169578453
C	-4.75707730781351	1.17602524111490	6.91173840584894
H	-5.77189023223627	1.57704401297770	6.92422559323613
H	-4.53822803532558	0.80430072226546	7.91349507667459
H	-4.06064222272748	1.98259984595096	6.67763794646192
P	-4.31134732629190	-0.69488327791229	0.38662518333234
H	-5.23641496109321	-1.66808690707127	0.84116502826308
H	-3.18728851891222	-1.36567305499961	0.93119296259357
O	-4.51302948090267	-0.14831268622564	3.05431525874986
P	-4.67742327143535	-0.24359243823863	5.71557088919709
H	-3.27163460292760	-0.43950469161525	5.76667124487834

***c*-C_2PS** E = -817.752472395206 au
ZPE = 0.06541592 au

C	-1.08416192265981	1.59679602809323	3.50560777370900
C	0.69295276051887	3.35206691630516	3.52563753264476
H	-1.11267852464915	0.98310713609438	2.60548884685936
H	-1.84641264027498	1.25222949543978	4.20069124796460
H	1.31714868382745	3.38315611449508	2.63285525782100
H	1.03888724624635	4.10215004968233	4.23317484374817
P	-1.13873063428324	3.43395118180672	3.10763580000697
H	-1.54740119993100	3.83285461273390	4.40728194515633
S	0.59883981120554	1.66188588534925	4.23748444208956

PHD$^{C\text{-}P}$-*c*-C_2PS E = -1199.742647382340 au
ZPE = 0.12108869 au

C	-4.53417559724042	0.40413068497549	1.58702156383904
H	-3.75118293387433	1.16467411673307	1.62919706671616

H	-5.49600095665228	0.91690189626555	1.51460428025574
C	-4.74542230725057	0.62936415924010	4.36152567802379
H	-3.98783255793906	1.41087598368026	4.27660599491796
H	-5.73309157030492	1.08054755439328	4.23828359935836
C	-4.72798585736343	1.35258721724986	7.09572024246140
H	-5.73204309622846	1.77945908199795	7.08040085029011
H	-4.50303576651084	1.07364784085238	8.12630627621877
H	-4.01466067363062	2.11119956938629	6.77089796026304
P	-4.29380688030546	-0.55485339284065	0.01225048930028
H	-5.21034508921106	-1.60004457020547	0.29634427941801
H	-3.16739578569070	-1.30868650861900	0.43249114452978
S	-4.48613410490213	-0.63035432167768	3.07499234426356
P	-4.70309868748408	-0.17722490327489	6.04390866017059
H	-3.30083902541149	-0.39449937815656	6.07738617997330

PHD$^{C\text{-}S}$-*c*-C_2PS E = -1255.977549066518 au
ZPE = 0.11254900 au

C	-3.90998535339049	0.20176758852597	0.84639132745090
H	-4.01021219930501	1.25529637009870	0.58277001679045
H	-4.41461450575898	0.01786336901566	1.79336373427039
C	-1.66605159586846	0.95403337486792	2.36686427661844
H	-2.46382521865130	0.99096718586791	3.11168270462274
H	-1.52777587049746	1.95286044806250	1.94648742773940
C	0.21909881859787	1.84306306503146	4.19607193967401
H	-0.58188810640538	1.98960385335774	4.92129412756172
H	1.14475362317240	1.63130992710195	4.73048863096358
H	0.35244412712222	2.74785224304081	3.60224916283750
S	-4.64119821548500	-0.83932162240364	-0.46836890631120
H	-5.92772074921001	-0.56242848895067	-0.19360129294888
S	-0.11340505062719	0.41056310004047	3.13514966514907
P	-2.08789755694018	-0.19173589622171	0.95586606840440
H	-2.22813181675292	-1.36862555743515	1.73491016717738
